# Use of Mutagenesis, Genetic Mapping and Next Generation Transcriptomics to Investigate Insecticide Resistance Mechanisms

**DOI:** 10.1371/journal.pone.0040296

**Published:** 2012-06-29

**Authors:** Predrag Kalajdzic, Stefan Oehler, Martin Reczko, Nena Pavlidi, John Vontas, Artemis G. Hatzigeorgiou, Charalambos Savakis

**Affiliations:** 1 Institute for Biological Research, University of Belgrade, Belgrade, Serbia; 2 Medical School, University of Crete, Heraklion, Greece; 3 Institute of Cellular and Developmental Biology, Biomedical Sciences Research Center “Alexander Fleming”, Varkiza, Greece; 4 Institute of Molecular Oncology, Biomedical Sciences Research Center “Alexander Fleming”, Varkiza, Greece; 5 Department of Biology, University of Crete, Heraklion, Greece; VIB & Katholieke Universiteit Leuven, Belgium

## Abstract

Insecticide resistance is a worldwide problem with major impact on agriculture and human health. Understanding the underlying molecular mechanisms is crucial for the management of the phenomenon; however, this information often comes late with respect to the implementation of efficient counter-measures, particularly in the case of metabolism-based resistance mechanisms. We employed a genome-wide insertional mutagenesis screen to *Drosophila melanogaster,* using a *Minos*-based construct, and retrieved a line (MiT[*w^−^*]3R2) resistant to the neonicotinoid insecticide Imidacloprid. Biochemical and bioassay data indicated that resistance was due to increased P450 detoxification. Deep sequencing transcriptomic analysis revealed substantial over- and under-representation of 357 transcripts in the resistant line, including statistically significant changes in mixed function oxidases, peptidases and cuticular proteins. Three P450 genes (Cyp4p2, Cyp6a2 and Cyp6g1) located on the 2R chromosome, are highly up-regulated in mutant flies compared to susceptible *Drosophila*. One of them (Cyp6g1) has been already described as a major factor for Imidacloprid resistance, which validated the approach. Elevated expression of the Cyp4p2 was not previously documented in *Drosophila* lines resistant to neonicotinoids. *In silico* analysis using the *Drosophila* reference genome failed to detect transcription binding factors or microRNAs associated with the over-expressed Cyp genes. The resistant line did not contain a *Minos* insertion in its chromosomes, suggesting a hit-and-run event, i.e. an insertion of the transposable element, followed by an excision which caused the mutation. Genetic mapping placed the resistance locus to the right arm of the second chromosome, within a ∼1 Mb region, where the highly up-regulated Cyp6g1 gene is located. The nature of the unknown mutation that causes resistance is discussed on the basis of these results.

## Introduction

Insecticide resistance is an increasing problem that compromises the control of insect pests of medical, veterinary and agricultural impact. An understanding of insecticide resistance mechanisms is essential for the subsequent development of tools and practices that can improve pest control interventions. During the last decades, extensive biochemical, genetic and molecular studies have been conducted to elucidate insecticide resistance mechanisms [1], [2], [3]. Knowledge of the mechanisms underlying target site resistance in major pests to some commonly used insecticides has been established to some extent [4], [5], [6]. The understanding of detoxification/metabolism-based insecticide resistance mechanisms has not kept similar pace, due to the complexity of the involved multi-gene systems and the lack of genome sequence data. However, in a few cases, the molecular basis of metabolism-based insecticide resistance mechanisms was identified. A single P450, CYP6P3, was over-expressed in pyrethroid resistant *Anopheles gambiae* mosquitoes, and it was capable of metabolizing pyrethroids [7]. Karunker et al. [8] showed that the *B*. *tabaci* cytochrome P450 *BTCYP6CM1* is capable of metabolizing the neonicotinoid Imidacloprid, one of the most important insecticides worldwide, and to confer neonicotinoid resistance.

These studies have shed light on cases of metabolism-based insecticide resistance mechanisms. However, there is a number of issues which remain unsolved, such as the underlying molecular mechanisms that are responsible for over-expression of detoxification enzymes. In addition, the information on molecular changes responsible for resistance often comes too late, i.e. when resistance has been irreversibly established in pest populations and/or when the active ingredient has already been replaced by others. The use of modern molecular approaches and models for the early identification or even prediction of insecticide resistance mechanisms could improve the management of the phenomenon.

*Drosophila melanogaster*, although not a pest species, has been used extensively for insecticide resistance research [9], [10], [11]. Resistance associated with the over-expression of a single P450 gene (Cyp6g1) has been documented for field-derived *Drosophila* lines resistant to Imidacloprid and DDT [12]. Over-expression correlated with the presence of a single insertion of an *Accord* transposable element into the 5′ end of the Cyp6g1 gene has also been reported [12]. A recent study of Cyp6g1 induction in transgenic *Drosophila* showed tissue-specific expression of this gene controlled by two distinct specific enhancers, suggesting that a single mutation event can modulate Cyp6g1 expression [13].

In contrast to field pest populations, which often possess a highly heterogeneous genetic background, the possibility for the generation of single mutations in a known and characterized background would substantially facilitate the identification of resistance-associated changes. Insertional mutagenesis using transposable elements has been an exceptionally efficient method to create mutants in phylogenetically very distant species, including *Drosophila melanogaster* [14]. The transposon *Minos*, a member of the Tc1/*mariner* superfamily, produces stable transformants with high efficiency in different insect species [15]. This allows genome-wide mutagenesis in insects [16] making *Minos* a promising genome-wide transgenesis tool.

High-throughput deep sequencing transcription profiling is a powerful approach to provide genome-wide information in a very short time and a cost effective way [17]. This method is classified as an “open” technology [18], which in contrast to “closed” technologies like microarrays, does not require biological or sequence information of the analyzed organism.

Here, by combining a genome-wide insertional mutagenesis screen and next generation transcriptomics, we were able to identify genes involved in Imidacloprid resistance in *Drosophila melanogaster* within a reasonable time frame and at moderate cost. Gene ontology analysis identified several overrepresented functional gene groups that are differentially expressed in the resistant *Drosophila* line. The results of our novel approach were in line with previous findings that showed that the Cyp6g1 gene is mainly responsible for resistance. The deep sequencing information was further explored to identify transcription binding factors or microRNAs possibly associated with the over-expression of Cyp genes, which are implicated in resistance. Genetic mapping placed the resistance locus to the right arm of the second chromosome, within a ∼1 Mb region in which the Cyp6g1 gene is located.

## Results

During the genome-wide insertional mutagenesis, about 12,900 new TREP insertions were generated. The *D. melanogaster* genome is estimated to contain approximately 13,000 known or predicted genes [19]. Using the information in Metaxakis et al. [16], it can be estimated that in our screen approximately 22% of known or predicted genes of *Drosophila* genome were hit at least once, directly or within 2 Kb upstream and downstream, excluding introns. Flies with new TREP insertions were selected on medium with 3 µg/ml of Imidacloprid (3 times higher than the LC99 of the susceptible line).

One female carrying a novel TREP insertion on the X-chromosome and exhibiting high resistance to Imidacloprid, was retrieved during the mutagenesis. Line MiT[*w* ^−^]3X, originating from the retrieved resistant female, was established. Genetic analysis of the MiT[*w* ^−^]3X line showed that the resistance trait mapped to the second chromosome (see below) but showed no linkage between the TREP insertion and the Imidacloprid resistance.

### Resistance to Imidacloprid and cross-resistance to DDT

The resistance was characterized by determining the susceptibility of the resistant mutant MiT[*w^−^*]3R2 (derived from MiT[*w^−^*]3X line) homozygous for the second chromosome and the control line iso31 to Imidacloprid. The Imidacloprid resistance of line MiT[*w^−^*]3R2 was found to be about 18-fold higher than that of the wild-type line iso31 (table 1). Mutant MiT[*w^−^*]3R2 showed resistance to Imidacloprid also at the adult stage, as well as cross resistance to DDT (table 1).

**Table 1:** LC50s for Imidacloprid and DDT of susceptible and resistant flies.

	IMIDACLOPRID		DDT	
	LC50 µg/ml (95% confidence limits)	RR*	LC50 µg/vial (95% confidence limits)	RR*
iso31	0.18 (0.15–0.21)	-	0.37 (0.15–0.65)	-
MiT[*w^−^*]3R2	3.30 (1.90–4.10)	18	37.50 (32.20–41.90)	100

* RR (resistance ratio) – LC50 value of the MiT[*w^−^*]3R2 line/LC50 value of the iso31 line.

### Biochemical assays

In order to assess if there is a contribution of known resistance pathways in resistance of mutant MiT[*w^−^*]3R2, the activities of cytochrome P450 monooxygenase, esterases and GSTs were analyzed (table 2). Esterase activity was measured using α- and β-naphthol, and GST activity was measured using 1-chloro-2,4-dinitrobenzene [20]. For both enzymes, no significant difference in activity was detected in the resistant line compared to line iso31. Cytochrome P450-dependent monooxygenase activity was determined by analyzing living third instar larvae, following the protocol of Inceoglu et al. [21]. The activity of cytochrome P450 was 3-fold higher in resistant MiT[*w^−^*]3R2 third instar larvae compared to third instar susceptible larvae (table 2).

**Table 2:** Activities of detoxification enzymes of resistant and susceptible lines.

	Cytochrome P450 monooxygenase (alive larvae) (pg/min/larvae) ± SD	Esterase (nmol a-naphthol produced/min/mg) ± SD	Esterase (nmol b naphthol produced/min/mg) ± SD	GST (μmole/min/mg) ± SD
MiT[*w^−^*]3R2	2.1±0.1	73±2	27±2	0.13±0.04
iso31	0.72±0.05	54±4	31±4	0.12±0.04
Fold difference MiT[*w^−^*] 3R2/iso31	3.0	1.4	0.8	1.0

### Deep sequencing analysis

Transcriptional profiling of the resistant MiT[*w^−^*]3R2 line was performed to obtain more information on the involvement of individual genes in the resistance. Deep sequencing yielded 16,344,712 and 16,859,384 mapped reads of 51 bp for MiT[*w^−^*]3R2 and iso31, respectively (GSM707197 MiT[*w^−^*]3R2 (GSM707197_Resistant_s_1_READS.txt.gz); GSM707198 iso31 (GSM707198_Susceptible_s_2_READS.txt.gz)). Alignment of the sequencing reads to the *Drosophila* reference genome (*Drosophila* release 5 sequence assembly Flybase) identified 18,963 transcripts for the susceptible line and 18,967 transcripts for the resistant line (GSE28560_total_number_of_transcripts.txt.gz). Using a minimum difference threshold of 2-fold, a total of 357 transcripts were found to be differently expressed between MiT[*w^−^*]3R2 and iso31 (GSE28560_resistant_vs._susceptible_UPREGULATED_GENES.txt.gz; GSE28560_resistant_vs._susceptible_DOWNREGULATED_GENES.txt.gz). In the resistant line, 150 genes were up-regulated and 207 genes were down-regulated (GSE28560_resistant_vs._susceptible_UPREGULATED_GENES.txt.gz; GSE28560_resistant_vs._susceptible_DOWNREGULATED_GENES.txt.gz).

Gene functional classification analysis by grouping genes based on functional similarities identified three functional groups in the up-regulated genes (table 3) and two functional groups in the down-regulated genes (table 4). The cytochrome P450 genes, proteolytic genes and genes showing peptidase activity were overrepresented in the up-regulated genes (table 3). Cuticular protein genes and genes showing peptidase activity were overrepresented in the down-regulated genes (table 4).

**Table 3:** Gene functional groups in the up-regulated genes (analyzed with the DAVID 6.7 BETA bioinformatics resource).

Peptidase activity, proteolysis		Cytochrome P450 (Cyp) genes		Metallocarboxypeptidase activity, biopolymer catabolic process, macromolecule catabolic process	
Enrichment Score: 5.10		Enrichment Score: 3.73		Enrichment Score: 2.32	
Gene name	Kappa*	Gene name	Kappa*	Gene name	Kappa*
CG31219	1.00	Cyp309a2	0.99	CG8539	0.89
Jonah 65Ai	1.00	Cyp6w1	0.99	CG8560	0.84
CG10469	1.00	Cyp4p2	0.96	CG15254	0.65
CG9676	1.00	Cyp6g2	0.96	CG2493	0.62
CG7829	0.97	Cyp4d14	0.94	CG31918	0.59
Jonah 25Biii	0.97	Cyp4e3	0.93		
CG32277	0.97	Cyp6g1	0.87		
CG4259	0.97	Cyp6a2	0.84		
Jonah 74E	0.94				
CG10477	0.94				
Jonah 25Bii	0.91				
CG11911	0.91				
CG4812	0.91				
Jonah 25Bi	0.84				
CG31918	0.59				
CG2493	0.56				

* Kappa score – The Kappa value quantitatively measures the degree to which genes share similar annotation terms (the higher the Kappa, the stronger the functional similarity).

Functional annotation clustering, which groups genes with similar predicted biological functions, identified 10 overrepresented groups in the up-regulated genes (table 5) and 13 overrepresented groups in the down-regulated genes (table 6). Among the functional groups overrepresented in the up-regulated genes, four clusters are connected to peptidase activity and three functional clusters are connected to P450 gene family activity (table 5). There were also other functional groups with significantly overrepresented members in the over-expressed genes, like oxidoreductase activity, mitotic sister chromatid segregation, electron carrier activity and response to DNA damage. In the down-regulated genes, groups like nutrient reservoir activity, chitin and aminoglycan metabolic processes, response to bacteria and immune response activity were identified (table 6).

**Table 4:** Gene functional groups in the down-regulated genes (analyzed with DAVID 6.7 BETA bioinformatics resource).

Structural constituent of chitin-based cuticle		Peptidase activity, proteolysis	
Enrichment Score: 2.52		Enrichment Score: 1.05	
Gene name	Kappa*	Gene name	Kappa*
CG1252	1.00	CG17234	1.00
CG2360	1.00	CG18180	0.97
CG2341	1.00	CG18179	0.97
Cuticular protein 56F	0.91	CG11037	0.94
Cuticular protein 47Ef	0.83	Jonah 66Ci	0.94
		Serine protease 12	0.88
		CG34043	0.80

* Kappa score – The Kappa value quantitatively measures the degree to which genes share similar annotation terms (the higher the Kappa, the stronger the functional similarity).

**Table 5:** Functional annotation clusters in the up-regulated genes (analyzed with the DAVID 6.7 BETA bioinformatics resource).

No. clusters 10							
Annotation Cluster 1	Enrichment Score: 5.62						
Category	Term	PValue	Gene name	Fold Enrichment	Bonferroni	Benjamini	FDR
GOTERM_MF_ALL	GO:0008236∼serine-type peptidase activity	2.92E-006	Jon25Bi, CG31219, CG11911, CG4259, Jon25Biii, Jon25Bii, CG2493, Ser8, CG10477, Jon65Ai, CG10469, Jon74E, CG7829, CG9676, CG32277, CG31219	4.69	5.81E-004	2.91E-004	3.66E-003
GOTERM_MF_ALL	GO:0017171∼serine hydrolase activity	3.15E-006	Jon25Bi, CG31219, CG11911, CG4259, Jon25Biii, Jon25Bii, CG2493, Ser8, CG10477, Jon65Ai, CG10469, Jon74E, CG7829, CG9676, CG32277, CG31219	4.66	6.27E-004	2.09E-004	3.95E-003
Annotation Cluster 2	Enrichment Score: 4.98						
Category	Term	PValue	Gene name	Fold Enrichment	Bonferroni	Benjamini	FDR
GOTERM_BP_ALL	GO:0006508∼proteolysis	2.13E-005	Jon25Bi, CG31219, CG11911, CG4259, Jon25Biii, Jon25Bii, CG31918, CG15254, CG2493, skpB, Ser8, CG10477, Jon65Ai, CG10469, CG8560, CG8539, Jon74E, CG7829, CG31704, CG9676, CG32277, CG31219	2.87	1.09E-002	1.09E-002	3.08E-002
GOTERM_BP_ALL	GO:0030163∼protein catabolic process	2.45E-005	Jon25Bi, CG31219, CG11911, CG4259, Jon25Biii, Jon25Bii, CG31918, CG15254, CG2493, skpB, Ser8, CG10477, Jon65Ai, CG10469, CG8560, CG8539, Jon74E, CG7829, CG31704, CG9676, CG32277, CG31219	2.84	1.26E-002	6.30E-003	3.54E-002
Annotation Cluster 3	Enrichment Score: 4.69						
Category	Term	PValue	Gene name	Fold Enrichment	Bonferroni	Benjamini	FDR
GOTERM_BP_ALL	GO:0043285∼biopolymer catabolic process	3.04E-005	Jon25Bi, CG31219, Ercc1, CG11911, CG4259, Jon25Biii, Jon25Bii, CG31918, CG15254, CG2493, skpB, Ser8, CG10477, Jon65Ai, CG10469, CG8560, CG8539, Jon74E, CG7829, CG31704, CG9676, CG32277, CG31219	2.70	1.56E-002	5.21E-003	4.39E-002
GOTERM_BP_ALL	GO:0009057∼macromolecule catabolic process	6.93E-005	Jon25Bi, CG31219, Ercc1, CG11911, CG4259, Jon25Biii, Jon25Bii, CG31918, CG15254, CG2493, skpB, Ser8, CG10477, Jon65Ai, CG10469, CG8560, CG8539, Jon74E, CG7829, CG31704, CG9676, CG32277, CG31219	2.55	3.51E-002	7.13E-003	1.00E-001
Annotation Cluster 4	Enrichment Score: 4.23						
Category	Term	PValue	Gene name	Fold Enrichment	Bonferroni	Benjamini	FDR
GOTERM_MF_ALL	GO:0070011∼peptidase activity, acting on L-amino acid peptides	5.34E-005	Jon25Bi, CG31219, CG11911, CG4259, Jon25Biii, Jon25Bii, CG31918, CG15254, CG2493, Ser8, CG10477, Jon65Ai, CG10469, CG8560, CG8539, Jon74E, CG7829, CG9676, CG32277, CG31219	2.95	1.06E-002	1.77E-003	6.69E-002
GOTERM_MF_ALL	GO:0008233∼peptidase activity	1.10E-004	Jon25Bi, CG31219, CG11911, CG4259, Jon25Biii, Jon25Bii, CG31918, CG15254, CG2493, Ser8, CG10477, Jon65Ai, CG10469, CG8560, CG8539, Jon74E, CG7829, CG9676, CG32277, CG31219	2.79	2.21E-002	2.23E-003	1.41E-001
Annotation Cluster 5	Enrichment Score: 4.12						
Category	Term	PValue	Gene name	Fold Enrichment	Bonferroni	Benjamini	FDR
GOTERM_MF_ALL	GO:0046906∼tetrapyrrole binding	8.58E-005	Cyp4e3, Cyp6a2, Cyp309a2, Cyp6w1, Cyp4p2, Cyp6g1, Cyp6g2, CG4009, Cyp4d14	6.26	1.69E-002	1.90E-003	1.07E-001
GOTERM_MF_ALL	GO:0020037∼heme binding	8.58E-005	Cyp4e3, Cyp6a2, Cyp309a2, Cyp6w1, Cyp4p2, Cyp6g1, Cyp6g2, CG4009, Cyp4d14	6.26	1.69E-002	1.90E-003	1.07E-001
Annotation Cluster 6	Enrichment Score: 3.04						
Category	Term	PValue	Gene name	Fold Enrichment	Bonferroni	Benjamini	FDR
GOTERM_BP_ALL	GO:0000070∼mitotic sister chromatid segregation	1.04E-003	rod, cid, Kmn1, Nuf2, Spc25	10.93	4.14E-001	4.75E-002	1.49E+000
GOTERM_BP_ALL	GO:0000819∼sister chromatid segregation	1.12E-003	rod, cid, Kmn1, Nuf2, Spc25	10.69	4.40E-001	4.72E-002	1.61E+000
Annotation Cluster 7	Enrichment Score: 2.99						
Category	Term	PValue	Gene name	Fold Enrichment	Bonferroni	Benjamini	FDR
GOTERM_BP_ALL	GO:0055114∼oxidation reduction	9.79E-004	CG31809, Cyp4e3, Cyp6a2, ry, Cp7Fb, CG11162, Cyp309a2, Cyp6w1, Cyp4p2, Cyp6g1, Cyp6g2, CG5235, CG4009, Fad2, Cyp4d14, CG31810	2.58	3.97E-001	5.46E-002	1.41E+000
GOTERM_MF_ALL	GO:0016491∼oxidoreductase activity	3.13E-003	CG31809, Cyp4e3, Cyp6a2, ry, Cp7Fb, CG11162, Cyp309a2, Cyp6w1, Cyp4p2, Cyp6g1, Cyp6g2, CG5235, CG4009, Fad2, Cyp4d14, CG31810	2.32	4.65E-001	5.07E-002	3.86E+000
Annotation Cluster 8	Enrichment Score: 2.88						
Category	Term	PValue	Gene name	Fold Enrichment	Bonferroni	Benjamini	FDR
GOTERM_MF_ALL	GO:0009055∼electron carrier activity	1.18E-003	Cyp4e3, Cyp6a2, ry, Cyp309a2, Cyp6w1, Cyp4p2, Cyp6g1, Cyp6g2, Cyp4d14	4.25	2.09E-001	2.11E-002	1.47E+000
Annotation Cluster 9	Enrichment Score: 2.79						
Category	Term	PValue	Gene name	Fold Enrichment	Bonferroni	Benjamini	FDR
GOTERM_BP_ALL	GO:0051310∼metaphase plate congression	3.83E-004	cid, Kmn1, Nuf2, Spc25	26.81	1.79E-001	2.78E-002	5.53E-001
GOTERM_BP_ALL	GO:0050000∼chromosome localization	4.68E-004	cid, Kmn1, Nuf2, Spc25	25.14	2.15E-001	2.97E-002	6.75E-001
GOTERM_BP_ALL	GO:0051303∼establishment of chromosome localization	4.68E-004	cid, Kmn1, Nuf2, Spc25	25.14	2.15E-001	2.97E-002	6.75E-001
GOTERM_BP_ALL	GO:0051656∼establishment of organelle localization	3.73E-002	cid, Kmn1, Nuf2, Spc25	5.36	1.00E+000	5.74E-001	4.23E+001
GOTERM_BP_ALL	GO:0051640∼organelle localization	4.80E-002	cid, Kmn1, Nuf2, Spc25	4.85	1.00E+000	6.53E-001	5.09E+001
Annotation Cluster 10	Enrichment Score: 2.17						
Category	Term	PValue	Gene name	Fold Enrichment	Bonferroni	Benjamini	FDR
GOTERM_BP_ALL	GO:0007067∼mitosis	3.73E-003	rod, Klp61F, Mcm2, cid, Kmn1, Nuf2, Spc25	4.63	8.55E-001	1.38E-001	5.27E+000
GOTERM_BP_ALL	GO:0000280∼nuclear division	3.98E-003	rod, Klp61F, Mcm2, cid, Kmn1, Nuf2, Spc25	4.57	8.72E-001	1.37E-001	5.61E+000
GOTERM_BP_ALL	GO:0000087∼M phase of mitotic cell cycle	3.98E-003	rod, Klp61F, Mcm2, cid, Kmn1, Nuf2, Spc25	4.57	8.72E-001	1.37E-001	5.61E+000
GOTERM_BP_ALL	GO:0048285∼organelle fission	4.65E-003	rod, Klp61F, Mcm2, cid, Kmn1, Nuf2, Spc25	4.43	9.10E-001	1.48E-001	6.52E+000

**Table 6:** Functional annotation clusters in the down-regulated genes (analyzed with the DAVID 6.7 BETA bioinformatics resource).

No clusters 13							
Annotation Cluster 1	Enrichment Score: 4.44						
Category	Term	PValue	Gene name	Fold Enrichment	Bonferroni	Benjamini	FDR
GOTERM_MF_FAT	GO:0045735∼nutrient reservoir activity	2.68E-005	Lsp1beta, Lsp1alpha, Lsp1gamma, CG11345	58.67	6.14E-003	6.14E-003	3.43E-002
GOTERM_MF_ALL	GO:0045735∼nutrient reservoir activity	3.21E-005	Lsp1beta, Lsp1alpha, Lsp1gamma, CG11345	55.50	8.99E-003	8.99E-003	4.25E-002
GOTERM_MF_1	GO:0045735∼nutrient reservoir activity	5.48E-005	Lsp1beta, Lsp1alpha, Lsp1gamma, CG11345	47.84	5.48E-004	5.48E-004	3.29E-002
Annotation Cluster 2	Enrichment Score: 3.66						
Category	Term	PValue	Gene name	Fold Enrichment	Bonferroni	Benjamini	FDR
GOTERM_BP_FAT	GO:0006026∼aminoglycan catabolic process	1.78E-004	Cht4, PGRP-SC1a, PGRP-SC1b, PGRP-SC2, Cht8, Cht9	17.16	8.50E-002	4.34E-002	2.55E-001
GOTERM_BP_FAT	GO:0000272∼polysaccharide catabolic process	2.01E-004	Cht4, PGRP-SC1a, PGRP-SC1b, PGRP-SC2, Cht8, Cht9	16.65	9.55E-002	3.29E-002	2.89E-001
GOTERM_BP_ALL	GO:0006026∼aminoglycan catabolic process	2.42E-004	Cht4, PGRP-SC1a, PGRP-SC1b, PGRP-SC2, Cht8, Cht9	15.89	1.39E-001	1.39E-001	3.59E-001
GOTERM_BP_ALL	GO:0000272∼polysaccharide catabolic process	2.74E-004	Cht4, PGRP-SC1a, PGRP-SC1b, PGRP-SC2, Cht8, Cht9	15.41	1.56E-001	8.13E-002	4.05E-001
Annotation Cluster 3	Enrichment Score: 2.68						
Category	Term	PValue	Gene name	Fold Enrichment	Bonferroni	Benjamini	FDR
GOTERM_MF_FAT	GO:0005214∼structural constituent of chitin-based cuticle	1.43E-003	Cpr49Ag, Cpr50Cb, Cpr56F, Ccp84Ad, Ccp84Ab, Ccp84Aa	5.60	2.81E-001	1.04E-001	1.82E+000
GOTERM_MF_ALL	GO:0005214∼structural constituent of chitin-based cuticle	1.97E-003	Cpr49Ag, Cpr50Cb, Cpr56F, Ccp84Ad, Ccp84Ab, Ccp84Aa	5.29	4.26E-001	1.69E-001	2.58E+000
GOTERM_MF_FAT	GO:0042302∼structural constituent of cuticle	2.23E-003	Cpr49Ag, Cpr50Cb, Cpr56F, Ccp84Ad, Ccp84Ab, Ccp84Aa	5.13	4.02E-001	1.21E-001	2.82E+000
GOTERM_MF_ALL	GO:0042302∼structural constituent of cuticle	3.06E-003	Cpr49Ag, Cpr50Cb, Cpr56F, Ccp84Ad, Ccp84Ab, Ccp84Aa	4.86	5.77E-001	1.94E-001	3.97E+000
Annotation Cluster 4	Enrichment Score: 2.59						
Category	Term	PValue	Gene name	Fold Enrichment	Bonferroni	Benjamini	FDR
GOTERM_BP_FAT	GO:0006022∼aminoglycan metabolic process	1.72E-003	Cht4, PGRP-SC1a, PGRP-SC1b, PGRP-SC2, Cht8, Cht9, CG7298, CG31077	5.38	5.77E-001	1.58E-001	2.45E+000
GOTERM_BP_FAT	GO:0005976∼polysaccharide metabolic process	2.50E-003	Cht4, PGRP-SC1a, PGRP-SC1b, PGRP-SC2, Cht8, Cht9, CG7298, CG31077	4.99	7.14E-001	1.64E-001	3.54E+000
GOTERM_BP_ALL	GO:0006022∼aminoglycan metabolic process	2.60E-003	Cht4, PGRP-SC1a, PGRP-SC1b, PGRP-SC2, Cht8, Cht9, CG7298, CG31077	4.98	8.01E-001	1.64E-001	3.79E+000
GOTERM_BP_ALL	GO:0005976∼polysaccharide metabolic process	3.75E-003	Cht4, PGRP-SC1a, PGRP-SC1b, PGRP-SC2, Cht8, Cht9, CG7298, CG31077	4.62	9.03E-001	2.08E-001	5.43E+000
Annotation Cluster 5	Enrichment Score: 2.49						
Category	Term	PValue	Gene name	Fold Enrichment	Bonferroni	Benjamini	FDR
GOTERM_BP_FAT	GO:0009617∼response to bacterium	8.09E-004	Dpt, AttC, Dro, DptB, TM9SF4, PGRP-SC1a, PGRP-SC1b	8.04	3.33E-001	9.62E-002	1.16E+000
GOTERM_BP_ALL	GO:0009617∼response to bacterium	1.17E-003	Dpt, AttC, Dro, DptB, TM9SF4, PGRP-SC1a, PGRP-SC1b	7.44	5.15E-001	1.65E-001	1.71E+000
GOTERM_BP_ALL	GO:0051707∼response to other organism	9.06E-003	Dpt, AttC, Dro, DptB, TM9SF4, PGRP-SC1a, PGRP-SC1b	4.62	9.96E-001	3.32E-001	1.26E+001
GOTERM_BP_ALL	GO:0009607∼response to biotic stimulus	1.22E-002	Dpt, AttC, Dro, DptB, TM9SF4, PGRP-SC1a, PGRP-SC1b	4.29	9.99E-001	3.78E-001	1.66E+001
Annotation Cluster 6	Enrichment Score: 2.40						
Category	Term	PValue	Gene name	Fold Enrichment	Bonferroni	Benjamini	FDR
GOTERM_MF_FAT	GO:0005344∼oxygen transporter activity	7.66E-003	Lsp1beta, Lsp1alpha, Lsp1gamma	22.00	8.29E-001	2.98E-001	9.39E+000
GOTERM_MF_ALL	GO:0005344∼oxygen transporter activity	8.59E-003	Lsp1beta, Lsp1alpha, Lsp1gamma	20.81	9.12E-001	3.84E-001	1.08E+001
Annotation Cluster 7	Enrichment Score: 2.11						
Category	Term	PValue	Gene name	Fold Enrichment	Bonferroni	Benjamini	FDR
GOTERM_BP_FAT	GO:0045087∼innate immune response	9.92E-003	Dpt, AttC, Dro, PGRP-SC1a, PGRP-SC1b, PGRP-SC2	5.84	9.93E-001	3.19E-001	1.34E+001
GOTERM_BP_ALL	GO:0045087∼innate immune response	1.30E-002	Dpt, AttC, Dro, PGRP-SC1a, PGRP-SC1b, PGRP-SC2	5.41	1.00E+000	3.64E-001	1.77E+001
Annotation Cluster 8	Enrichment Score: 1.99						
Category	Term	PValue	Gene name	Fold Enrichment	Bonferroni	Benjamini	FDR
GOTERM_BP_FAT	GO:0006955∼immune response	4.33E-003	Dpt, AttC, Dro, DptB, TM9SF4, PGRP-SC1a, PGRP-SC1b, PGRP-SC2	4.47	8.86E-001	1.95E-001	6.05E+000
GOTERM_BP_ALL	GO:0006955∼immune response	6.41E-003	Dpt, AttC, Dro, DptB, TM9SF4, PGRP-SC1a, PGRP-SC1b, PGRP-SC2	4.14	9.82E-001	2.83E-001	9.11E+000
GOTERM_BP_ALL	GO:0002376∼immune system process	1.95E-002	Dpt, AttC, Dro, DptB, TM9SF4, PGRP-SC1a, PGRP-SC1b, PGRP-SC2	3.25	1.00E+000	4.40E-001	2.53E+001
GOTERM_BP_1	GO:0002376∼immune system process	2.09E-002	Dpt, AttC, Dro, DptB, TM9SF4, PGRP-SC1a, PGRP-SC1b, PGRP-SC2	3.19	3.59E-001	1.99E-001	1.51E+001
Annotation Cluster 9	Enrichment Score: 1.88						
Category	Term	PValue	Gene name	Fold Enrichment	Bonferroni	Benjamini	FDR
GOTERM_BP_FAT	GO:0019731∼antibacterial humoral response	1.74E-003	Dpt, AttC, Dro, DptB	16.28	5.81E-001	1.35E-001	2.47E+000
GOTERM_BP_ALL	GO:0019731∼antibacterial humoral response	2.19E-003	Dpt, AttC, Dro, DptB	15.07	7.43E-001	1.76E-001	3.19E+000
GOTERM_BP_FAT	GO:0019730∼antimicrobial humoral response	2.47E-002	Dpt, AttC, Dro, DptB	6.28	1.00E+000	5.43E-001	3.03E+001
GOTERM_BP_ALL	GO:0019730∼antimicrobial humoral response	3.04E-002	Dpt, AttC, Dro, DptB	5.81	1.00E+000	5.49E-001	3.67E+001
GOTERM_BP_FAT	GO:0006959∼humoral immune response	3.83E-002	Dpt, AttC, Dro, DptB	5.29	1.00E+000	6.62E-001	4.30E+001
GOTERM_BP_ALL	GO:0006959∼humoral immune response	4.66E-002	Dpt, AttC, Dro, DptB	4.90	1.00E+000	6.80E-001	5.08E+001
Annotation Cluster 10	Enrichment Score: 1.83						
Category	Term	PValue	Gene name	Fold Enrichment	Bonferroni	Benjamini	FDR
GOTERM_MF_FAT	GO:0030247∼polysaccharide binding	1.31E-002	Cht4, PGRP-SC1a, PGRP-SC1b, PGRP-SC2, Cht8, CG7298, CG31077	4.22	9.52E-001	3.96E-001	1.55E+001
GOTERM_MF_FAT	GO:0001871∼pattern binding	1.31E-002	Cht4, PGRP-SC1a, PGRP-SC1b, PGRP-SC2, Cht8, CG7298, CG31077	4.22	9.52E-001	3.96E-001	1.55E+001
GOTERM_MF_ALL	GO:0001871∼pattern binding	1.67E-002	Cht4, PGRP-SC1a, PGRP-SC1b, PGRP-SC2, Cht8, CG7298, CG31077	3.99	9.91E-001	5.45E-001	2.00E+001
GOTERM_MF_ALL	GO:0030247∼polysaccharide binding	1.67E-002	Cht4, PGRP-SC1a, PGRP-SC1b, PGRP-SC2, Cht8, CG7298, CG31077	3.99	9.91E-001	5.45E-001	2.00E+001
Annotation Cluster 11	Enrichment Score: 1.81						
Category	Term	PValue	Gene name	Fold Enrichment	Bonferroni	Benjamini	FDR
GOTERM_BP_FAT	GO:0006032∼chitin catabolic process	1.11E-002	Cht4, Cht8, Cht9	18.31	9.96E-001	3.28E-001	1.48E+001
GOTERM_BP_ALL	GO:0006032∼chitin catabolic process	1.29E-002	Cht4, Cht8, Cht9	16.95	1.00E+000	3.77E-001	1.75E+001
GOTERM_MF_FAT	GO:0004568∼chitinase activity	1.89E-002	Cht4, Cht8, Cht9	13.89	9.87E-001	4.65E-001	2.17E+001
GOTERM_MF_ALL	GO:0004568∼chitinase activity	2.11E-002	Cht4, Cht8, Cht9	13.15	9.97E-001	5.75E-001	2.46E+001
Annotation Cluster 12	Enrichment Score: 1.53						
Category	Term	PValue	Genes	Fold Enrichment	Bonferroni	Benjamini	FDR
GOTERM_BP_FAT	GO:0055114∼oxidation reduction	8.91E-003	Nos, CG14946, Gld, Cyp9b1, Prx2540-2, Cyp4p1, CG32557, CG6762, Cyp4d21, CG18231, CG17843, CG30093, Eip71CD	2.29	9.89E-001	3.11E-001	1.21E+001
GOTERM_BP_ALL	GO:0055114∼oxidation reduction	1.67E-002	Nos, CG14946, Gld, Cyp9b1, Prx2540-2, Cyp4p1, CG32557, CG6762, Cyp4d21, CG18231, CG17843, CG30093, Eip71CD	2.12	1.00E+000	4.06E-001	2.21E+001
Annotation Cluster 13	Enrichment Score: 1.48						
Category	Term	PValue	Gene name	Fold Enrichment	Bonferroni	Benjamini	FDR
GOTERM_MF_ALL	GO:0004553∼hydrolase activity, hydrolyzing O-glycosyl compounds	3.34E-002	Cht4, Mal-B1, Mal-A7, Cht8, Cht9	4.08	1.00E+000	6.53E-001	3.62E+001
GOTERM_MF_ALL	GO:0016798∼hydrolase activity, acting on glycosyl bonds	4.58E-002	Cht4, Mal-B1, Mal-A7, Cht8, Cht9	3.68	1.00E+000	6.98E-001	4.62E+001

Eight of the 150 up-regulated genes, were members of the P450 gene family (GSE28560_resistant_vs._susceptible_UPREGULATED_GENES.txt.gz). The three highest over-expressed P450 genes, with more than 15-fold expression in MiT[*w^−^*]3R2 were Cyp4p2 (100-fold), Cyp6a2 (19.9-fold) and Cyp6g1 (16.3-fold) (GSE28560_resistant_vs._susceptible_UPREGULATED_GENES.txt.gz).

We performed quantitative real time PCR to validate the expression difference of two representative cytochrome P450 genes (Cyp6g1 and Cyp6a2), already known to play important role in insecticide resistance. Cyp6g1 showed an expression difference between the resistant and susceptible lines of 8.4 (±0.7), while gene Cyp6a2 showed an expression difference of 10.3 (± 1.7) fold. The expression difference of Cyp4p2 was also validated with quantitative PCR, and showed 4.9 (± 0.3) fold elevated expression in the resistant line.

### Chromosomal mapping of the Imidacloprid-resistance locus

**Figure 1:**
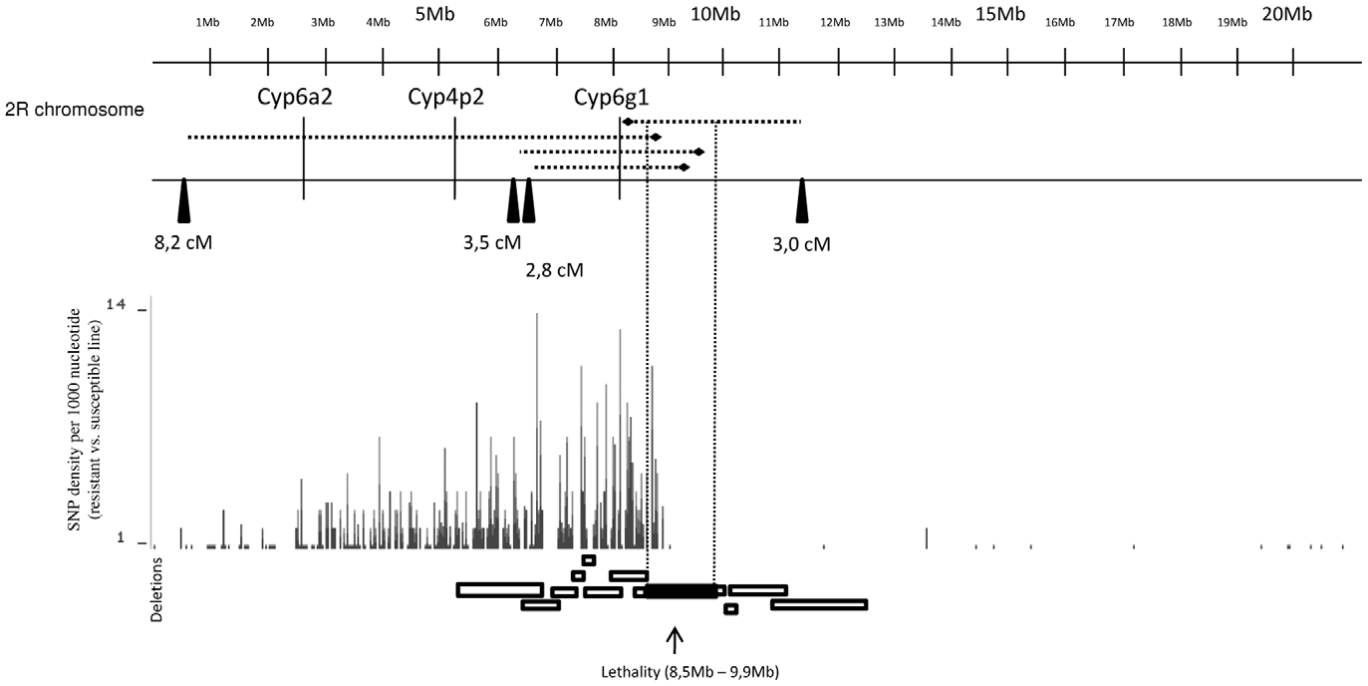
Mapping of the lethality and resistance loci on the right arm of the second chromosome in the resistant line MiT[*w^−^*]3R2. The resistance locus was mapped relative to P element insertions to a region between 8 Mb and 8.5 Mb (black arrows on the second scale, distance between insertion and resistance region is indicated with dotted horizontal lines). The location of the three highly expressed P450 genes (Cyp6a2, Cyp6g1 and Cyp4p2) in the resistant MiT[*w^−^*]3R2 line is indicated. Below is a comparison of single nucleotide polymorphism (SNP) density (per 1 Kb) between resistant line MiT[*w^−^*]3R2 and susceptible line iso31. At the bottom, Bloomington deletions overlapping lethality locus (filled box) and flanking the lethality locus (open boxes) (lethality maps to the region between 8.5 Mb and 9.9 Mb, close to the place of recombination).

Mapping of the resistance locus of MiT[*w^−^*]3X flies to a chromosome was done with standard genetic tools. Males from Imidacloprid-resistant line MiT[*w^−^*]3X were individually crossed with *w*1118iso/Dp(1;Y)y+; nocSco/SM6a females, who carry chromosome 2 balancer SM6a. Progeny from this cross, heterozygous for the second chromosome, was selected on Imidacloprid as described. Both resistant male and female progeny emerged, implying that the resistance locus does not map to the sex chromosome. Resistant heterozygous male progeny carrying SM6a was individually crossed with iso31 (susceptible line) females. Progeny from this cross was also selected on Imidacloprid. None of the progeny carried the chromosome 2 balancer, which places the resistance on the second chromosome. Equivalent crosses were performed between resistant MiT[*w^−^*]3X males and *w*1118/Dp(1;Y)y+; TM2/TM6C, Sb1 female flies, carrying two balancers of chromosome 3. This cross showed that there is no correlation between the resistance locus and the third chromosome, confirming that the resistance locus is located on the second chromosome. Line MiT[*w^−^*]3R2/CyO, which lacked the *Minos* insertion but carried the resistance trait and a lethal locus was derived from MiT[w]3X. The lethality locus was mapped to the right arm of the second chromosome, using the Bloomington Stock Center *Drosophila* deletion kit, between position 49C1-4; 50C23-D2 (8.5 Mb –9.9 Mb; figure 1). Recombination mapping placed the lethality locus on the same chromosome arm that harbors the resistance locus. Line MiT[*w^−^*]3R2, homozygous for the second chromosome resistance locus, was established during the recombination analysis.

MiT[*w^−^*]3R2 was derived from the original resistant line (MiT[*w^−^*]3X) using *Drosophila* lines with different genetic backgrounds (TREP 2.30 and BOEtTA have a yw background, while [SM6a, MiT 2.4]/Sco] is an iso31 derivative). In order to replace the genetic background of the resistant mutant with that of the susceptible control line, MiT[*w^−^*]3R2 was back-crossed with iso31 for 6 generations under selection with 3 µg/ml of Imidacloprid.

### P element mapping

In order to narrow down the position of the resistance locus in the MiT[*w^−^*]3R2 line on the 2R chromosome, genetic mapping relative to P element insertions was used. The distance between a P element located at ∼0.5 Mb and the resistance locus was determined to be 8.2 cM. The distance between a P element located at ∼6.1 Mb and the resistance locus is 3.5 cM. The distance between a P element located at ∼ 6.5 Mb and the resistance locus was determined to be 2.8 cM. The distance between a P element located at ∼ 11.2 Mb and the resistance locus was determined to be 3.0 cM. These genetic distances were converted into Mb using estimates of local recombination rates according to Fiston-Lavier et al. [22] and Singh et al. [23] (figure 1). The genetic mapping relative to the P element insertions places the resistance locus roughly between 8 Mb and 9.7 Mb on the right arm of the second chromosome (figure 1) in the MiT[*w^−^*]3R2 mutant line.

### In silico analysis

A comparison of single nucleotide polymorphisms (SNP) of the deep sequencing data between the resistant line MiT[*w^−^*]3R2 and the susceptible line iso31 was done for chromosome 2R (figure 1). Resistant line MiT[*w^−^*]3R2 had been back-crossed with line iso31 under selection with 3 µg/ml of Imidacloprid in order to homogenize the genetic background. The SNP comparison indicates a hybrid origin of the 2R chromosome, where the right half comes from iso31, while the left half comes from a different line, most likely yw (figure 1). This result indicates a recombination event on 2R, close to the region between 8.5 Mb and 9.9 Mb, to which the lethality was mapped (figure 1). The position of the lethality, resistance and recombination break point shows that the recombination event occurred between the resistance and lethality loci (figure 1). The highly over-expressed Cyp6g1 gene (16.3-fold – deep sequencing analysis; 8.4-fold- real time PCR analysis) lies close to the recombination break point to the region into which the resistance locus has been placed (figure 1).

Comparison of the sequences of the Cyp genes differently expressed in the resistant versus the susceptible line showed no sequence changes of the P450 proteins. We also analyzed the flanking sequences of differentially expressed genes for possible common transcription factor binding sites. *In silico* analysis, using the JASPAR database [24], did not detect common transcription binding factors either for the subgroup of Cyp genes or for all over-expressed genes.

Similarly, a search for predicted targets of microRNAs, performed with DIANA-microT version 3.0 [25], in the 3′UTRs of all up-regulated and down-regulated genes, or of just the up-regulated and down-regulated Cyp genes, did not identify any significantly overrepresented common target sites.

## Discussion

We tested a combined approach of mutagenesis and next generation transcriptomics to study insecticide resistance in the model organism *Drosophila melanogaster*.

A *Minos*-based construct was used for genome-wide insertional gene activation mutagenesis. During this screen, an Imidacloprid-resistant *D. melanogaster* female was retrieved. In the resistant line MiT[*w^−^*]3R2/CyO lacking the TREP (*Minos*) insertion, which was derived from this retrieved mutant female, both lethality and resistance were detected. In a recombination experiment resistance was separated from lethality and line MiT[*w^−^*]3R2 homozygous for resistant second chromosome was established. This line was further analyzed for the resistance mechanism.

Cross-resistance of the Imidacloprid-selected MiT[*w^−^*]3R2 mutant to DTT (table 1) suggests metabolic resistance as the mechanism of resistance in this line. Furthermore, biochemical analysis showed increased P450 activity in the resistant line compared to the susceptible line (table 2).

The Illumina parallel short-sequencing technology was used to obtain total cDNA sequences of the resistant line and of the non-resistant isogenic line iso31 (*w* ^1118^_iso_; 2_iso_; 3_iso_) [26]. This approach was used in order to identify and quantify differences in expression between mutant line MiT[*w^−^*]3R2 and susceptible line iso31, covering nearly all *D. melangaster* genes. Out of 357 genes differently expressed in the resistance line, 150 were up-regulated, and 207 genes were down-regulated in comparison to the susceptible line.

Gene ontology functional classification of the sequenced transcripts identified a significantly up-regulated P450 family group and two groups of genes coding for peptidase activity in the resistant line. Significantly overrepresented down-regulated groups of genes were cuticular protein genes and other peptidase genes.

Deep sequencing analysis detected eight members of the P450 family, Cyp4p2, Cyp6a2, Cyp6g1, Cyp6w1, Cyp4e3, Cyp309a2, Cyp6g2 and Cyp4d14, with elevated expression in the resistant line. Genes encoding glutathione-S-transferases, as well as esterases did not show elevated expression in the resistant line. The most highly over-expressed P450 genes as detected with deep sequencing and confirmed by real time PCR, are Cyp4p2, Cyp6a2 and Cyp6g1 (GSE28560_resistant_vs._susceptible_UPREGULATED_GENES.txt.gz). The cytochrome P450 genes play an important role in insecticide resistance, because of their variety and the broad substrate specificity of some P450 genes [27]. We report for the first time elevated expression of the Cyp4p2 gene in a *D. melanogaster* line resistant to Imidacloprid and DDT, although its role in resistance (if any) remains to be elucidated. The detoxification function of Cyp6a2 and Cyp6g1 in *Drosophila* is well documented. Over-expression of Cyp6g1 in *Drosophila* confers resistance to DDT and neonicotinoids [12], [28], [29] which provides a certain degree of validation in our approach for detecting genes conferring insecticide resistance. The Cyp6a2 is also highly expressed in different insecticide resistant *Drosophila* strains [30], [31], [32], [33] and the CYP6A2 encoded enzyme can metabolize insecticides [34], [35].

Five other P450 genes (Cyp6w1, Cyp4e3, Cyp309a2, Cyp6g2 and Cyp4d14) detected in the resistant line are over-expressed up to 6-fold. Microarray analysis showed that expression of Cyp6w1 is higher in DDT resistant *Drosophila* strain compared to a susceptible line [33]. Over-expression of the Cyp6g2 gene confers resistance to diazonin and nitenpyram in transgenic *Drosophila* [28]. To date, Cyp4e3, Cyp309a2 and Cyp4d14 have not been implied in insecticide resistance.

A number of cuticular protein genes were down-regulated in the resistant mutant compared to the susceptible line (table 4). This could occur as a result of the general stress response induced by the up-regulated detoxification system. It is not likely that the down-regulation of cuticular protein genes plays a role in the insecticide resistance mechanism. It would be in disaccord with the fact that reduced cuticular penetration of insecticides can contribute to resistance in some insect species [36], [37], [38].

The identification of a group of 21 up-regulated genes involved in peptidase activity is consistent with the finding that genes coding for peptidase activity are also significantly over-expressed in DDT resistant *Drosophila* [33]. The role of the proteolytic genes and genes showing peptidase activity in insecticide resistance is still poorly understood. There is increasing evidence of involvement of protein metabolism in insecticide resistances of different insect species [39], [40], [41], [42], [43]. Proteases may be involved in modification of enzyme conformation and protein biosynthesis, in order to meet energy requirements during xenobiotic stress [39].

Other groups of overrepresented members among the up- or down-regulated genes belong to the following categories: oxidoreductase activity, chromosome establishment, organelle localization and cellular response to DNA damage (up-regulated; table 5) and nutrient reservoir activity, response to bacteria, biotic stimulus and immune response (down-regulated; table 6). Down-regulation of genes involved in immune response was not seen in other DDT-resistant *Drosophila* lines [33]. Oxidoreductase activity plays a role in detoxification, while the other biological processes could be an indication of general stress response.

The line MiT[*w^−^*]3R2, homozygous for the resistance chromosome, derives from the mutant line MiT[*w^−^*]3R2/CyO heterozygous for the second chromosome carrying both resistance and lethality. Genetic analysis of the mutant line MiT[*w^−^*]3R2/CyO line placed the lethality locus to the region between 8.5 and 9.9 Mb on the right arm of the second chromosome of *D. melanogaster*. Single nucleotide polymorphism analysis between the resistant line homozygous for resistant chromosome MiT[*w^−^*]3R2 and the susceptible line iso31 indicates that the recombination event that separated the lethality from the resistance locus occurred in close vicinity to the lethality locus (figure 1). The three highest up-regulated P450 genes Cyp4p2, Cyp6a2 and Cyp6g1 are also located on the right arm of the second chromosome, but they are not closely linked (figure 1). Mapping against P element insertions confirmed that the resistance locus lies on the right arm of the second chromosome between 8 Mb and 9.7 Mb. Chromosomal mapping of the resistance in DDT and Imidacloprid resistant *Drosophila* lines [44] placed the DDT resistance locus (*Rst (2) DDT*) in an area that overlaps the interval in which the resistance locus of MiT[*w^−^*]3R2 is located. The position of the lethality locus (between 8.5 and 9.9 Mb), together with the SNP analysis and P element mapping, suggests that the resistance locus in MiT[*w^−^*]3R2 lies within an interval of less than ∼1 Mb (figure 1). Interestingly, the highly up-regulated Cyp6g1 (16.3-fold – deep sequencing analysis; 8.4-fold – real time PCR analysis) gene is located within this range. In the mentioned study of Daborn and colleagues [44] the Cyp6g1 is strongly suggested as the main candidate gene responsible for the resistance in DDT and Imidacloprid resistant *Drosophila* lines.

The mutation event which causes the resistance in MiT[*w^−^*]3R2 remains to be identified. The resistance locus is not linked to an insertion of the transposon used in the screen. It is conceivable that a “hit and run” *Minos* insertion effect might be responsible for the mutation, where the transposon first integrated and then re-excised. In *Drosophila*, *Minos* often leaves behind upon excision either a characteristic six bp “footprint” or a deletion around the site of insertion [45], both of which can be mutagenic. It has been suggested that mutations of *trans*-regulating factor/s, or of cis-acting elements of some of the Cyp genes are responsible for insecticide resistance in *Drosophila* [46], [47], [48]. A recent report suggests that a single mutation event in a specific enhancer can modulate Cyp6g1 tissue-specific induction in *Drosophila* flies [13]. One might thus speculate that a single mutation event occurred in a *cis*-acting element of the Cyp6g1 gene, increasing the expression of this gene. This in turn could activate a resistance cascade, affecting the expression of other Cyp genes involved in resistance. Alternatively, the mutation might involve a gene encoding a transcription factor or a microRNA which regulates in *trans* the Cyp genes involved. We have so far no evidence for the latter assumption, since an *in silico* search failed to identify common transcription factor motifs regulating the over-expressed P450 genes. The same is true for common predicted microRNA targets in the 3′UTRs. Additional analyses are required in order to pinpoint the exact cause of resistance in the MiT[*w^−^*]3R2 mutant.

## Materials and Methods

### Drosophila lines

**Figure 2:**
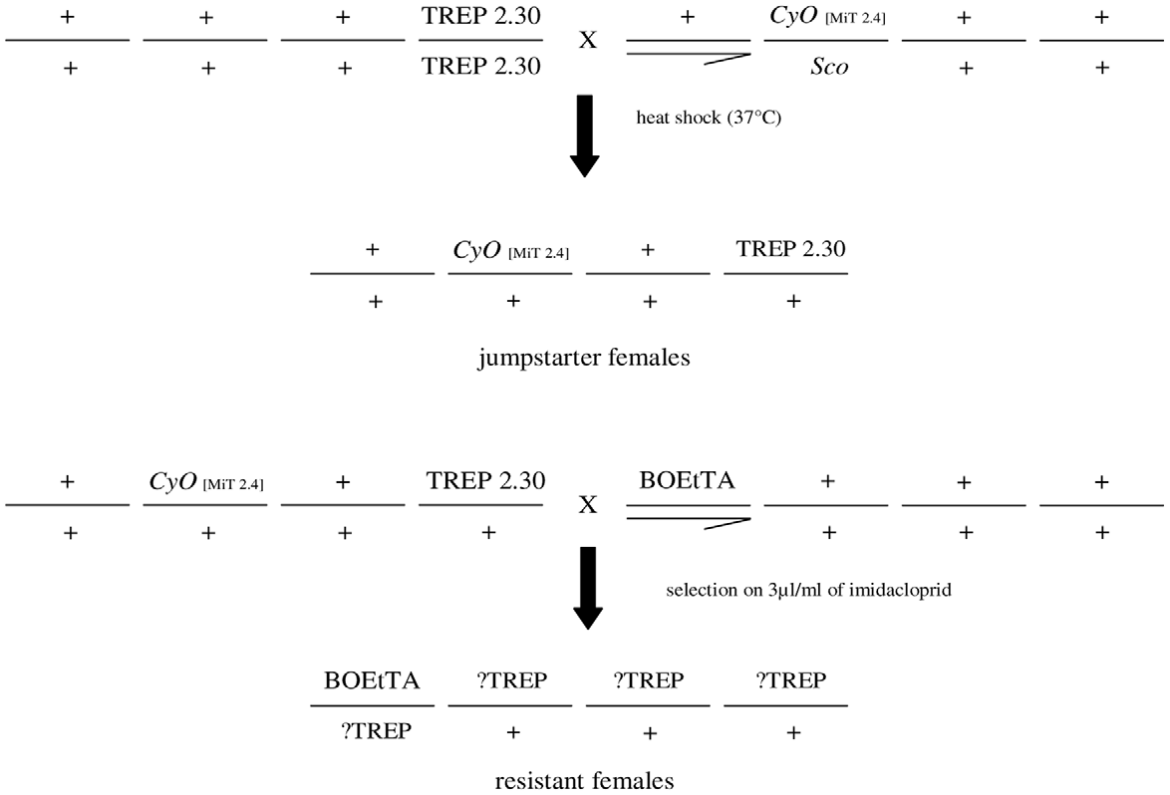
Crossing scheme of the genome-wide insertional mutagenesis system. TREP 2.30 – promoter delivery, minimal promoter under tTA control, *w^+^* marker *CyO* [MiT 2.4] – *Minos* transposase source, CyO marker *Sco* – Sco marker BOEtTA – tTA source, *egfp* marker.

*D. melanogaster* stocks were maintained on standard cornmeal-agar-yeast medium at 24°C with a 12-hour light/12-hour dark cycle. We analyzed a *Drosophila melanogaster* line (MiT[*w^−^*]3R2) resistant to the neonicotinoid Imidacloprid, retrieved during a transposon *Minos*-based insertional mutagenesis screen. Three transgenic *Drosophila* lines were used for *Minos*-based insertional mutagenesis: TREP 2.30, BOEtTA [49] and an iso31 derivative [SM6a, MiT 2.4]/Sco] [16]. Line 2.30 carries a single insertion of the *Minos* transposon TREP, which contains a minimal promoter driven by the tetO operator. When inserted next to or into a gene, TREP can cause over-expression of the gene in the presence of the tetracycline trans-activator tTA [50, Kiupakis, Oehler and Savakis, manuscript in preparation]. Line BOEtTA carries a single insertion of a *Minos* transposon which produces tTA [49]. The mobilization of the TREP construct and generation of flies with new insertions was performed with a standard “jumpstarter” system [51] (figure 2). Flies with new TREP insertions were selected for insecticide resistance during egg to adult development on medium with Imidacloprid. We aimed to generate highly resistant flies, thus mutagenized flies were selected on a concentration of Imidacloprid 3 times higher than the LC99 of susceptible line iso31 (3µg/ml). Female individuals carrying both TREP 2.30 and BOEtTA construct were scored for resistance (figure 2). The insertional site distribution of new TREP insertions was estimated according to Metaxakis et al. [16]. The correction for multiple insertions into the same genes was done using the Poisson distribution, assuming the same probability of recovering insertions for all loci [52]. One resistant female with a *Minos* insertion located on the X chromosome was retrieved from the screen and further analyzed.

For the genetic analysis, we also used balancer lines for the second chromosome, *w* ^1118^_iso_/Dp(1;Y)y^+^; noc^Sco^/SM6a [26], and for the third chromosome, *w* ^1118^_iso_/Dp(1;Y)y^+^; TM2/TM6C, Sb^1^ [26]. Isogenic line iso31 (*w* ^1118^_iso_; 2_iso_; 3_iso_) [26] was used as an insecticide susceptible control line (wild-type).

### Bioassays

The insecticide Imidacloprid (98.7%, Bayer CropScience GmbH) was added directly to the standard medium for the screening experiments. Per vial, 50 eggs were added, and egg-to-adult viability was determined. Six different concentrations were used with 8 replicas per concentration.

A contact assay was used for Imidacloprid and DDT assay. 35 ml glass vials were coated with DTT (DDT 4,4′– DDT PESTANAL®, SIGMA-ALDRICH) on the inside by evaporating 200 µl of acetone containing the required amounts of DDT. Coated vials were plugged with cotton wool soaked with 5% sucrose. The mortality of 25 flies (1–3 days old) per vial was scored after 24 hours. Each DTT amount was assayed for sex different concentration with 4 replicas per concentration.

All toxicology data were analyzed for LC50 (lethal concentration 50), using Probit statistics [53] with SPSS 10.0 for Windows [54].

### Biochemical assays

The activities of esterases, GSTs and cytochrome P450 dependent monooxygenases were determined as previously described [20], [21]. The activity of cytochrome P450 dependent monooxygenases was measured in live third instar larvae. For all assays, activity was measured at 25°C on a SpectraMax M2 microplate reader and quantified with the integrated software SoftMax pro v5.

### Deep sequencing analysis

Deep sequencing data were deposited to the GEO site (series record GSE28560). The following URL allows review of the record. http://www.ncbi.nlm.nih.gov/geo/query/acc.cgi?token=nbyhvegqykeoanm&acc=GSE28560.

Total RNA from 3 day old *Drosophila melanogaster* adults was extracted using standard Trizol RNA isolation protocol (http://quantgen.med.yale.edu/). Preparation of cDNA for sequencing was done with the Illumina mRNA Seq V2 kit (Illumina, Inc). Formation of single molecule arrays, cluster growth and sequencing were done according to the standard protocols from Illumina, Inc. Sequencing was performed with a 2008 Illumina Genome Analyzer, version 2 (GA2). Mapping of the 51 nucleotides (nt) long sequencing reads of both lines, MiT[*w^−^*]3R2 and iso31, to the reference genome (*Drosophila* release 5 sequence assembly Flybase) was performed with software RMAP, version 2.05 [55]. Genes with 10 or less reads for one line and 50 reads or less for the other line were excluded from further analysis. The minimum difference threshold between lines was set to 2-fold. Analysis of up-regulated and down-regulated genes was performed with the DAVID 6.7 BETA release bioinformatics resources [56].

### Quantitative Real Time PCRs

Total RNA from *D. melanogaster* 1–3 day old adult flies was extracted using same protocol as for deep sequencing analysis. Synthesis of first-strand cDNA and PCR reactions were performed on a Techne TC-412 PCR machine. Synthesis of the First-Strand cDNA was done with the AccuScript® High Fidelity RT-PCR System. Expression of Cyp6g1 and Cyp6a2 was measured relative to the housekeeping ribosomal protein gene Rp49. For this purpose, three sets of primers were designed. In order to obtain products specific for the cDNA templates, primers were designed to span exon-intron junctions. The forward and reverse primer sequences were as follows: Cyp6g1 – 5′ACCCTTATGCAGGAGATTG3′ and 5′TAGGCTGTTAGCACGAATG3′; Cyp6a2 – 5′GTTACTGCCTGTATGAGTTGG3′ and 5′TAGAGCCTCAGGGTTTCTG3′; Rp49 – 5′CGGTTACGGATCGAACAAGCG3′ and 5′TTGGCGCGCTCGACAATCT3′. Quantitative real time PCR was performed using the QIAGEN SYBR green kit with the DNA Engine Opticon TM MJ Research analyzer. Three technical replicates were performed on each of three biological samples. The efficiency of PCR amplification with each gene-specific primer pair was analyzed with five serial dilutions in three technical replicates. Cycling conditions were: 94°C for 5 min, then 37 cycles of 94°C for 30 sec, 52°C for 30 sec and 72°C for 30 sec (plate reading at 78°C, 80°C and 82°C). Data were analyzed with the MJ Opticon Monitor 3.1 analysis software. Calculations were done with software REST-MCS [57]. Additionally, relative expression of the Cyp4p2 in the resistant line was analyzed. Quantitative real time PCR for Cyp4p2 was performed using same protocols as for Cyp6g1 and Cyp6a2 expression analysis. Flies maintained for more than 25 generations on standard medium after deep sequencing and Cyp6g1 and Cyp6a2 expression analysis, were used for Cyp4p2 expression analysis. The forward and reverse primer sequences were as follows: Cyp4p2 – 5′ CTGAAAAGGCATCCTTACGC 3′ and 5′ TTGGGATCGATAACAGGCAG 3′. Quantitative real time PCR was performed on the Bio-Rad CFX analyzer with cycling conditions: 95°C for 2 min, then 35 cycles of 95°C for 15 sec, 55°C for 30 sec and 60°C for 30 sec (melt curve 60 to 95 C, increment 1.0 C).

### Mapping of the lethality

We used the Bloomington Stock Center *Drosophila* deletion kit (111 lines) for the second chromosome to map the lethality locus (text S1). Individual crosses were set up between MiT[*w^−^*]3R2/CyO flies carrying a balancer of the second chromosome (SM6a/lethality) and the lines from the deletion kit (also carrying a balancer of the second chromosome). The progeny was scored for the presence of the balancer chromosome.

### P element mapping of the resistance locus

To narrow down the resistance locus on the right arm of the second chromosome, four lines with P elements insertions were employed (Bloomington *Drosophila* Stock Center, IU; text S2). The resistant MiT[*w^−^*]3R2 flies do not carry any visible marker gene, while the flies carrying P element insertions have *w^+^* as phenotypic marker. Resistant flies were mass crossed with flies carrying the P element insertion. Virgin female progeny with red eyes (one chromosome deriving from the resistant line and the other from the P element line) were collected and crossed with iso31 males. For each experiment, 50 female flies, heterozygous for the resistance chromosome were crossed with 25 iso31 males, per replica. Each experiment had eight replicas with a total number of 250 females for each P element line. After 2–3 days, crossed flies were transferred to medium with 3 µg/ml of Imidacloprid. Progeny was scored for recombination events. At least 1000 emerged flies were analyzed per replica. Recombination rates were calculated as the ratios of the total number of recombinant flies over the total number of emerged flies. The distance between the P element insertions and resistance was calculated in centimorgans (cM), from which the physical distance was calculated using estimates of the local recombination rates at the sites of the P element insertions after Fiston-Lavier et al. [22] and Singh et al. [23]. This estimate was not possible for one of the P elements, which is too close (about 0.5 Mb) to the centromere. Here, the recombination rate for the interval between the P element and the average position of the resistance locus, as determined relative to the other three P elements, was calculated.

### In silico analysis

A comparison of single nucleotide polymorphisms (SNP) of the deep sequence data between the resistant line MiT[*w^−^*]3R2 and the susceptible line iso31 was done for the right arm of the second chromosome. Genomic SNP analysis of the pooled assembly of the resistant and the susceptible strains reads were done with the Gigabayes SNP discovery algorithm (improved PolyBayes algorithm [58]) and MOSAIC algorithm [59], using all Refseq mRNA transcripts of the dm3 assembly [60] as a reference. A polymorphism probability threshold of 0.9 is used, with alleles requiring a minimal overall coverage of 10 and of 5 for the minor allele. A SNP density track with the number of SNPs in 1 Kb tiling windows was created. The SNP density was visualized with the UCSC Genome Browser on *D*. *melanogaster* release 5 (http://genome.ucsc.edu/) [61] and is presented for chromosome 2R.

The *in silico* search for overrepresented transcription factor binding sites was conducted using the JASPAR database [24]. All up-regulated and down-regulated genes, as well as the subset of Cyp genes (up-regulated, down-regulated and all), were analyzed for the presence of transcription factor binding sites. The sequence of all genes was retrieved from Flybase (*Drosophila* release 5 sequence assembly) [62]. Regions from of 3Kb upstream to1Kb downstream of the gene start and the 3′UTR sequences were analyzed.

A survey of predicted targets of microRNAs in the 3′UTR of all up-regulated and down-regulated genes, as well as the subsets of up-regulated and down-regulated Cyp genes, was performed with DIANA-microT (version 3.0) [25].

We also compared the sequences of Cyp genes differently expressed in the resistant versus the susceptible line for nucleotide differences. Comparison of the DNA sequences and translation to amino acids were done with the APE software (http://biologylabs.utah.edu/jorgensen/wayned/ape/).

## Supporting Information

Text S1 **Deletion kit stocks (second chromosome).**
(DOC)

Text S2 **P element stocks.**
(DOC)

## References

[1] Hemingway J (2000). The molecular basis of two contrasting metabolic mechanisms of insecticide resistance.. Insect Biochem Mol Biol.

[2] Oakeshott JG, Horne I, Sutherland TD, Russell RJ (2003). The genomics of insecticide resistance.. Genome Biology.

[3] ffrench-Constant RH, Daborn PJ, Le Goff G (2004). The genetics and genomics of insecticide resistance.. Trends Genet.

[4] ffrench-Constant RH, Anthony N, Aronstein K, Rocheleau T, Stilwell G (2000). Cyclodiene insecticide resistance: from molecular to population genetics.. Annu Rev Entomol.

[5] Hemingway J, Hawkes NJ, McCarroll L, Ranson H (2004). The molecular basis of insecticide resistance in mosquitoes.. Insect Biochem Mol Biol.

[6] Soderlund DM (2008). Pyrethroids, knockdown resistance and sodium channels.. Pest Manag Sci.

[7] Müller P, Warr E, Stevenson BJ, Pignatelli PM, Morgan JC (2008). Field-caught permethrin-resistant Anopheles gambiae overexpress CYP6P3, a P450 that metabolises pyrethroids.. PLoS Genet.

[8] Karunker I, Morou E, Nikou D, Nauen R, Sertchook R (2009). Structural model and functional characterization of the Bemisia tabaci CYP6CM1vQ, a cytochrome P450 associated with high levels of imidacloprid resistance.. Insect Biochem Mol Biol.

[9] Wilson TG (1988). Drosophila melanogaster (Diptera: Drosophilidae): a model insect for insecticide resistance studies.. Journal of Economic Entomology.

[10] Wilson TG (2001). Resistance of Drosophila to toxins.. Annu Rev Entomol.

[11] Perry T, Batterham P, Daborn PJ (2011). The biology of insecticidal activity and resistance.. Insect Biochem Mol Biol.

[12] Daborn PJ, Yen JL, Bogwitz MR, Le Goff G, Feil E (2002). A single P450 allele associated with insecticide resistance in Drosophila.. Science.

[13] Chung H, Boey A, Lumb C, Willoughby L, Batterham P (2011). Induction of a detoxification gene in Drosophila melanogaster requires an interaction between tissue specific enhancers and a novel cis-regulatory element.. Insect Biochem Mol Biol.

[14] Ivics Z, Izsvak Z (2010). The expanding universe of transposon technologies for gene and cell engineering.. Mob DNA.

[15] Pavlopoulos A, Oehler S, Kapetanaki MG, Savakis C (2007). The DNA transposon *Minos* as a tool for transgenesis and functional genomic analysis in vertebrates and invertebrates.. *Genome Biology*.

[16] Metaxakis A, Oehler S, Klinakis A, Savakis C (2005). *Minos* as a genetic and genomic tool in *Drosophila melanogaster*. Genetics..

[17] Lister R, Gregory BD, Ecker JR (2009). Next is now: new technologies for sequencing of genomes, transcriptomes, and beyond. Curr Opin Plant Biol..

[18] Green CD, Simons JF, Taillon BE, Lewin DA (2001). Open systems: panoramic views of gene expression.. J Immunol Methods.

[19] Adams MD, Celniker SE, Holt RA, Evans CA, Gocayne JD (2000). The genome sequence of Drosophila melanogaster.. Science Mar 24.

[20] Taskin V, Küçükakyüz K, Arslan T, Çöl B, Taşkın BG (2007). The biochemical basis of insecticide resistance and determination of esterase enzyme patterns by using page in field collected populations of Drosophila melanogaster from Muğla province of Turkey. J. Cell Mol. Biol..

[21] Inceoglu AB, Waite TD, Christiansen JA, Mcabee RD, Kamita SG (2009). A Rapid Luminescent Assay for Measuring Cytochrome P450 Activity in Individual Larval *Culex pipiens* Complex Mosquitoes (Diptera: Culicidae). J. Med. Entomol..

[22] Fiston-Lavier AS, Singh ND, Lipatov M, Petrov DA (2010). Drosophila melanogaster recombination rate calculator.. Gene.

[23] Singh ND, Arndt PF, Petrov DA (2005). Genomic heterogeneity of background substitutional patterns in Drosophila melanogaster.. Genetics.

[24] Wasserman WW, Sandelin A (2004). Applied bioinformatics for the identification of regulatory elements. Nat Rev Genet..

[25] Maragkakis M, Alexiou P, Papadopoulos GL, Reczko M, Dalamagas T (2009). Accurate microRNA target prediction correlates with protein repression levels.. *BMC Bioinformatics* Sep 18.

[26] Ryder E, Blows F, Ashburner M, Bautista-Llacer R, Coulson D (2004). The DrosDel collection: a set of P-element insertions for generating custom chromosomal aberrations in Drosophila melanogaster.. Genetics.

[27] Scott JG, Kasai S (2004). Evolutionary plasticity of monooxygenase-mediated resistance.. Pesticide Biochemistry and Physiology.

[28] Daborn PJ, Lumb C, Boey A, Wong W, ffrench-Constant RH (2007). Evaluating the insecticide resistance potential of eight *Drosophila melanogaster* cytochrome P450 genes by transgenic over-expression.. Insect Biochem and Mol Biol.

[29] Chung H, Bogwitz MR, McCart C, Andrianopoulos A, ffrench-Constant RH (2007). Cis regulatory elements in the Accord retrotransposon result in tissue-specific expression of the *Drosophila melanogaster* insecticid resistance gene Cyp6g1.. Genetics.

[30] Waters LC, Zelhof AC, Shaw BJ, Cha'ng L-Y (1992). Possible involvement of the long terminal repeat of transposable element 17.6 in regulating expression of an insecticide resistance-associated P450 gene in *Drosophila*. Proc. Natl. Acad. Sci.. U S A.

[31] Maitra S, Dombrowski SM, Waters LC, Ganguly R (1996). Three second chromosome-linked clustered Cyp6 genes show differential constitutive and barbital-induced expression in DDT-resistant and susceptible strains of Drosophila melanogaster.. Gene.

[32] Dombrowski SM, Krishnan R, Witte M, Maitra S, Diesing C (1998). Constitutive and barbital-induced expression of the Cyp6a2 allele of a high producer strain of CYP6A2 in the genetic background of a low producer strain.. Gene.

[33] Pedra JH, McIntyre LM, Scharf ME, Pittendrigh BR (2004). Genome-wide transcription profile of field- and laboratory-selected dichlorodiphenyltrichloroethane (DDT)-resistant Drosophila.. Proc Natl Acad Sci U S A.

[34] Dunkov BC, Guzov VM, Mocelin G, Shotkoski F, Brun A (1997). The Drosophila cytochrome P450 gene Cyp6a2: structure, localization, heterologous expression, and induction by Phenobarbital. DNA Cell Biol..

[35] Saner C, Weibel B, Wurgler FE, Sengstag C (1996). Metabolism of promutagens catalyzed by Drosophila melanogaster CYP6A2 enzyme in Saccharomyces cerevisiae. Environ. Mol. Mutagen..

[36] Scott JG, Georghiou GP (1986a). The biochemical genetics of permethrin resistance in the Learn-PyR strain of house fly. Biochem. Genet..

[37] Scott JG, Georghiou GP (1986b). Mechanisms responsible for high levels of permethrin resistance in the house fly. Pestic. Sci..

[38] Apperson CS, Georghiou GP (1975). Mechanisms of resistance to organophosphorus insecticides in Culex tarsalis.. J Econ Entomol.

[39] Ahmed S, Wilkins RM, Mantle D (1998). Comparison of proteolytic enzyme activities in adults of insecticide resistant and susceptible strains of the housefly M. domestica L. Insect Biochem Mol Biol.

[40] Mushtaq A S, Richard WM, David M, Shakoori AR (2003). Effect of starvation on proteases in insecticide-resistant and susceptible strains of Tribolium castaneum.. Pakistan journal of zoology.

[41] Araujo RA, Guedes RN, Oliveira MG, Ferreira GH (2008). Enhanced activity of carbohydrate- and lipid-metabolizing enzymes in insecticide-resistant populations of the maize weevil, Sitophilus zeamais.. Bull Entomol Res.

[42] Lopes KV, Silva LB, Reis AP, Oliveira MG, Guedes RN (2010). Modified alpha-amylase activity among insecticide-resistant and -susceptible strains of the maize weevil, Sitophilus zeamais.. J Insect Physiol.

[43] Silva LB, Reis AP, Pereira EJ, Oliveira MG, Guedes RN (2010). Partial purification and characterization of trypsin-like proteinases from insecticide-resistant and -susceptible strains of the maize weevil, Sitophilus zeamais.. Comp Biochem Physiol B Biochem Mol Biol.

[44] Daborn P, Boundy S, Yen J, Pittendrigh B, ffrench-Constant R (2001). DDT resistance in Drosophila correlates with Cyp6g1 over-expression and confers cross-resistance to the neonicotinoid imidacloprid.. Mol Genet Genomics.

[45] Arcà B, Zabalou S, Loukeris T, Savakis C (1997). Mobilization of a *Minos* transposon in *Drosophila melanogaster* chromosomes and chromatid repair by heteroduplex formation.. *Genetics*.

[46] Maitra S, Dombrowski S, Basu M, Raustol O, Waters L (2000). Factors on the third chromosome affect the level of Cyp6a2 and Cyp6a8 expression in Drosophila melanogaster.. Gene.

[47] Morra R, Kuruganti S, Lam V, Lucchesi JC, Ganguly R (2010). Functional analysis of the cis-acting elements responsible for the induction of the Cyp6a8 and Cyp6g1 genes of Drosophila melanogaster by DDT, phenobarbital and caffeine.. Insect Molecular Biology.

[48] Giraudo M, Unnithan GC, Le Goff G, Feyereisen R (2010). Regulation of cytochrome P450 expression in Drosophila: Genomic insight.. Pest Biochem and Physiol.

[49] Koukidou M, Klinakis A, Reboulakis C, Zagoraiou L, Tavernarakis N (2006). Germ line transformation of the olive fly *Bactrocera oleae* using a versatile transgenesis marker. Insect Mol Biol..

[50] Gossen M, Bonin AL, Bujard H (1993). Control of gene activity in higher eukaryotic cells by prokaryotic regulatory elements.. Trends in biochemical sciences.

[51] Cooley L, Kelley R, Spradling A (1988). Insertional mutagenesis of the Drosophila genome with single P elements.. Science.

[52] Pollock DD, Larkin JC (2004). Estimating the degree of saturation in mutant screens.. Genetics.

[53] Finney DJ (1971). Probit analysis. London: Cambridge Univ. Press.. 333 p.

[54] SPSS Inc (1999). SPSS Base 10.0 for Windows User's Guide. SPSS Inc.. Chicago IL.

[55] Smith AD, Xuan Z, Zhang MQ (2008). Using quality scores and longer reads improves accuracy of solexa read mapping.. BMC Bioinformatics.

[56] Huang DW, Sherman BT, Lempicki RA (2009). Systematic and integrative analysis of large gene lists using DAVID Bioinformatics Resources. Nature Protoc..

[57] Pfaffl MW, Horgan GW (2001). Calculation Software for the Relative Expression in real time PCR using Pair Wise Fixed Reallocation Randomisation Test.. Nucleic Acids Research Vol 29.

[58] Marth GT, Korf I, Yandell MD, Yeh RT, Gu Z (1999). A general approach to single-nucleotide polymorphism discovery.. Nat Genet.

[59] Gonzalez JR, Rodriguez-Santiago B, Caceres A, Pique-Regi R, Rothman N (2011). A fast and accurate method to detect allelic genomic imbalances underlying mosaic rearrangements using SNP array data.. BMC Bioinformatics.

[60] Pruitt KD, Tatusova T, Klimke W, Maglott DR (2009). NCBI Reference Sequences: current status, policy and new initiatives.. Nucleic Acids Res 37(Database issue).

[61] Kent WJ, Sugnet CW, Furey TS, Roskin KM, Pringle TH (2002). The human genome browser at UCSC.. Genome Res.

[62] Tweedie S, Ashburner M, Falls K, Leyland P, McQuilton P (2009). FlyBase: enhancing Drosophila Gene Ontology annotations.. Nucleic Acids Res 37(Database issue).

