# Supplementary material for: Use of Mutagenesis, Genetic Mapping and Next Generation Transcriptomics to Investigate Insecticide Resistance Mechanisms
Source: PLoS One. 2012 Jun 29;7(6):e40296. doi: 10.1371/journal.pone.0040296 (PMC3386967; doi:10.1371/journal.pone.0040296)
Supplement: Text S1 — Deletion kit stocks (second chromosome). (DOC) [file pone.0040296.s001.doc]

Deletion kit stocks (second chromosome)

**Stock Number: 90**

**Genotype:** Df(2L)C144, dpp[d-ho] ed[1]/In(2LR)Gla, wg[Gla-1] Bc[1] Egfr[E1]

**Chromosome(s):** 2

**Breakpts/Insertion:** 22F4-23A1;23C2-4 (R5.1 estimated cytology)

**Date added:** 3/30/1995 **Donor:** Bill Gelbart

**Comments:** DK2

**Stock Number: 140**

**Genotype:** y[1] w[67c23]; Df(2L)Trf-C6R31/CyO

**Chromosome(s):** 2

**Breakpts/Insertion:** 28DE (within)

**Date added:** 10/24/1995 **Donor:** Tom Crowley

**Comments:** DK2

**Stock Number: 167**

**Genotype:** Df(2L)TW161, cn[1] bw[1]/CyO

**Chromosome(s):** 2

**Breakpts/Insertion:** 38A6-B1;40A4-B1

**Date added:** 12/1/1988 **Donor:** Ted Wright

**Comments:** DK2; M[-] pr[-], poor Cy expression in stock

**Stock Number: 179**

**Genotype:** In(1)w[m4h], y[1]; Df(2L)TE29Aa-11, dp[*]/CyO

**Chromosome(s):** 1;2

**Breakpts/Insertion:** 28E4-7;29B2-C1

**Date added:** 10/27/1995 **Donor:** Steve Beckendorf **Donor's source:** Gunter Reuter

**Comments:** DK2

**Stock Number: 190**

**Genotype:** Df(2R)en-A/CyO

**Chromosome(s):** 2

**Breakpts/Insertion:** 47D3;48B2

**Date added:** 11/10/1995 **Donor:** MaryAnn Martin **Donor's source:** Umea Stock Center

**Comments:** DK2; intact copy of this deficiency, fails to complement Df(2R)en28, Df(2R)en-SFX31, Df(2R)en-B, and Df(2R)en30, MA.M.; poor Cy expression, K.M. 2/26/97

**Stock Number: 198**

**Genotype:** w[118]; Df(2R)H3C1/CyO

**Chromosome(s):** 1;2

**Breakpts/Insertion:** 43F;44D3-8

**Date added:** 2/2/1996 **Donor:** Ken Howard

**Comments:** DK2; distal breakpoint should coincide with or overlap proximal breakpoint of Df(2R)H3E1, K.H. 12/15/95; w[118] is not a typo, K.M. 2/2/96

**Stock Number: 201**

**Genotype:** w[118]; Df(2R)H3E1/CyO

**Chromosome(s):** 1;2

**Breakpts/Insertion:** 44D1-4;44F12

**Date added:** 2/2/1996 **Donor:** Ken Howard

**Comments:** DK2; proximal breakpoint should coincide with or overlap distal breakpoint of Df(2R)H3C1, K.H. 12/15/95; w[118] is not a typo, K.M. 2/2/96

**Stock Number: 282**

**Genotype:** Dp(1;Y)y[+]/y[1]; Df(2R)X58-12/SM5

**Chromosome(s):** 1;Y;2

**Breakpts/Insertion:** 58D1-2;59A

**Date added:** 2/2/1996 **Donor:** Terry Orr-Weaver

**Comments:** DK2; deficiency includes M(2)58F, SM5 carries a duplication for this region, adult viability is poor without the duplication, T. O.-W. 5/5/95; poor Cy expression, K.M. 2/26/06

**Stock Number: 420**

**Genotype:** Df(2L)TW137, cn[1] bw[1]/CyO, Dp(2;2)M(2)m[+]

**Chromosome(s):** 2

**Breakpts/Insertion:** 36C2-4;37B9-C1, 36F + ?

**Date added:** 12/1/1988 **Donor:** Ted Wright

**Comments:** DK2

**Stock Number: 442**

**Genotype:** Df(2R)CX1, wg[12] b[1] pr[1]/SM1

**Chromosome(s):** 2

**Breakpts/Insertion:** 49C1-4;50C23-D2

**Date added:** 4/1/1993 **Donor:** Nick Baker

**Comments:** DK2. Carlos Flores reported that PCR showed full length and shorter P elements in stock, K.C. 4/01.

**Stock Number: 490**

**Genotype:** In(1)w[m4]; Df(2L)E110/CyO

**Chromosome(s):** 1;2

**Breakpts/Insertion:** 3C1-2;20F, 25F3-26A1;26D3-11

**Date added:** 3/14/1996 **Donor:** Kenneth Tartof

**Comments:** DK2; balancer shown as Cy, CyO is a guess, K.M. 3/14/96

**Stock Number: 567**

**Genotype:** Df(2L)pr-A16, cn[1] bw[1]/CyO

**Chromosome(s):** 2

**Breakpts/Insertion:** 37B2-12;38D2-5

**Date added:** 12/1/1988 **Donor:** Ted Wright

**Comments:** DK2. Poor Cy expression, K.C. 9/05.

**Stock Number: 693**

**Genotype:** Df(2L)sc19-8/SM6b; Dp(2;1)B19, y[1], ed[1] dp[o2] cl[1]

**Chromosome(s):** 2

**Breakpts/Insertion:** 24C2-8;25C8-9, 24D4;25F2;9B-C

**Date added:** 12/1/1988 **Donor:** Janos Szidonya

**Comments:** DK2. Poor Cy[1] expression, K.C.

**Stock Number: 739**

**Genotype:** Df(2R)M41A4/SM1

**Chromosome(s):** 2

**Breakpts/Insertion:** 41A;41A

**Date added:** 4/1/1987 **Donor:** Caltech Stock Center

**Comments:** DK2

**Stock Number: 749**

**Genotype:** In(2R)bw[VDe2L]Cy[R]/In(2LR)Gla, wg[Gla-1]

**Chromosome(s):** 2

**Breakpts/Insertion:** h42-h43;42A2-3 + 58A4;59E1 (Df + Dp)

**Date added:** 4/1/1987 **Donor:** Caltech Stock Center

**Comments:** DK2

**Stock Number: 754**

**Genotype:** Df(2R)vg-C/CyO, P{ry[+t7.2]=sevRas1.V12}FK1

**Chromosome(s):** 2

**Breakpts/Insertion:** 49A4-13;49E7-F1

**Date added:** 4/1/1987 **Donor:** Caltech Stock Center

**Comments:** DK2; proximal breakpoint information from P. Adler, cited as personnal communication in DIS 71:154, K.M. 8/29/95; rebalanced to rescue, w[-] floating, K.M. 2/23/01

**Stock Number: 757**

**Genotype:** y[1] w[*]/Dp(1;Y)y[+]; Df(2R)P34/CyO

**Chromosome(s):** 1;Y;2

**Breakpts/Insertion:** 55E2-4;56C1-11

**Date added:** 8/19/1996 **Donor:** Winifred Doane **Donor's source:** Dan Moore

**Comments:** DK2

**Stock Number: 781**

**Genotype:** Df(2L)cl-h3/SM6b

**Chromosome(s):** 2

**Breakpts/Insertion:** 25D2-4;26B2-5

**Date added:** 12/1/1988 **Donor:** Janos Szidonya

**Comments:** DK2

**Stock Number: 1007**

**Genotype:** Df(2R)nap9/Dp(2;2)BG, In(2LR)Gla, wg[Gla-1]

**Chromosome(s):** 2

**Breakpts/Insertion:** 42A1-2;42E6-F1, 41A-B;42BC

**Date added:** 8/26/1995 **Donor:** Barry Ganetzky

**Comments:** DK2

**Stock Number: 1045**

**Genotype:** Df(2L)Mdh, cn[1]/Dp(2;2)Mdh3, cn[1]

**Chromosome(s):** 2

**Breakpts/Insertion:** 30D-30F;31F, 30D1-E1;32D1-32F3

**Date added:** 4/1/1987 **Donor:** Caltech Stock Center **Donor's source:** E.H. Grell

**Comments:** DK2; dark eye color present, L.C.; assumes MdhA = Mdh, K.M.; deficiency heterozygotes should have a moderate Minute phenotype, K.M.7/28/98

**Stock Number: 1145**

**Genotype:** Dp(1;Y)B[S]; Df(2R)en30/SM5

**Chromosome(s):** 2

**Breakpts/Insertion:** 48A3-4;48C6-8

**Date added:** 4/1/1987 **Donor:** Caltech Stock Center **Donor's source:** Sue Eberlein

**Comments:** DK2

**Stock Number: 1491**

**Genotype:** Df(2L)r10, cn[1]/CyO

**Chromosome(s):** 2

**Breakpts/Insertion:** 35D1;36A6-7

**Date added:** 5/1/1991 **Donor:** Michael Ashburner **Donor's source:** Seigfreid Roth

**Comments:** DK2; X-ray excision of ry[+] insert at 36A, J.R.; ry[*] floating?, K.M.

**Stock Number: 1547**

**Genotype:** Df(2R)PC4/CyO

**Chromosome(s):** 2

**Breakpts/Insertion:** 55A;55F

**Date added:** 11/1/1987 **Donor:** Jose Bonner **Donor's source:** Trudi Schupbach or Umea

**Comments:** DK2

**Stock Number: 1567**

**Genotype:** Df(2L)JS17, dpp[d-ho]/CyO, P{ry[+t7.2]=en1}wg[en11]

**Chromosome(s):** 1;2

**Breakpts/Insertion:** 23C1-2;23E1-2

**Date added:** 3/30/1995 **Donor:** Bill Gelbart

**Comments:** DK2; aka Df(2L)179.17, deletes Mad and gammaTub23CD, W.G.; blue balancer, identity probable but not confirmed, K.M. 12/13/95; z[1] w[11E4] floating, K.M. 2/26/97

**Stock Number: 1682**

**Genotype:** Df(2R)or-BR6, cn[1] bw[1] sp[1]/In(2LR)lt[G16L]bw[V32gR], bw[V32g]

**Chromosome(s):** 2

**Breakpts/Insertion:** 59D5-10;60B3-8 (Df); 59E;60E5-8 (Dp on In)

**Date added:** 10/1/1993 **Donor:** Michael Ashburner **Donor's source:** Bruce Reed

**Comments:** DK2

**Stock Number: 1702**

**Genotype:** Df(2R)X1, Mef2[X1]/CyO, Adh[nB]

**Chromosome(s):** 2

**Breakpts/Insertion:** 46C;47A1

**Date added:** 7/1/1994 **Donor:** Martha O'Brien

**Comments:** DK2; cytology of M. Burg via M. O'Brien, K.M.

**Stock Number: 1743**

**Genotype:** w[1118]; Df(2R)B5, px[1] sp[1]/CyO, Adh[nB]

**Chromosome(s):** 1;2

**Breakpts/Insertion:** 46A;46C

**Date added:** 7/1/1994 **Donor:** Martha O'Brien

**Comments:** DK2; small possibility balancer is SM6, generated as excision of P{A}N21, M.O.

**Stock Number: 1888**

**Genotype:** Df(2R)ST1, Adh[n5] pr[1] cn[*]/CyO

**Chromosome(s):** 2

**Breakpts/Insertion:** 42B3-5;43E15-18

**Date added:** 11/1/1987 **Donor:** Jose Bonner **Donor's source:** Trudi Schupbach

**Comments:** DK2; M. Ashburner says this is the correct cytology according to both his notes and Genetics 135:105, K.M. 1/25/96

**Stock Number: 2414**

**Genotype:** w[*]; Df(2L)spd[j2], wg[spd-j2]/CyO, P{ry[+t7.2]=ftz/lacB}E3

**Chromosome(s):** 1;2

**Breakpts/Insertion:** 27C1-2;28A

**Date added:** 12/2/1996 **Donor:** Shelagh Campbell **Donor's source:** Steve Cohen

**Comments:** DK2; blue balancer, but specific identity of construct unknown, this is a guess, K.M. 3/2/99

**Stock Number: 2471**

**Genotype:** Df(2R)M60E/In(2LR)bw[V32g], bw[V32g]

**Chromosome(s):** 2

**Breakpts/Insertion:** 60E2-3;60E11-12

**Date added: Donor:**

**Comments:** DK2

**Stock Number: 2583**

**Genotype:** Df(2L)cact-255rv64, cact[chif64]/CyO; ry[506]

**Chromosome(s):** 2;3

**Breakpts/Insertion:** 35F-36A;36D

**Date added:** 12/2/1996 **Donor:** John Tower

**Comments:** DK2

**Stock Number: 2604**

**Genotype:** Df(2R)Px2/CyO, P{ry[+t7.2]=sevRas1.V12}FK1

**Chromosome(s):** 2

**Breakpts/Insertion:** 60C5-6;60D9-10

**Date added:** 4/1/1987 **Donor:** Thom Kaufman

**Comments:** DK2; w[1118] segregating, K.M. 11/17/06

**Stock Number: 2892**

**Genotype:** Df(2L)N22-14/CyO

**Chromosome(s):** 2

**Breakpts/Insertion:** 29C1-2;30C8-9

**Date added:** 4/25/1997 **Donor:** Michael Ashburner

**Comments:** DK2

**Stock Number: 3079**

**Genotype:** Df(2L)Prl, Prl[1] nub[Prl]/CyO

**Chromosome(s):** 2

**Breakpts/Insertion:** 32F1-3;33F1-2

**Date added:** 11/1/1987 **Donor:** Jose Bonner **Donor's source:** Trudi Schupbach

**Comments:** DK2

**Stock Number: 3084**

**Genotype:** Df(2L)ast2/SM1

**Chromosome(s):** 2

**Breakpts/Insertion:** 21D1-2;22B2-3

**Date added:** 11/1/1987 **Donor:** Jose Bonner **Donor's source:** Trudi Schupbach

**Comments:** DK2

**Stock Number: 3133**

**Genotype:** Df(2L)dp-79b, dp[DA] cn[1]/In(2LR)bw[V1], b[1] bw[V1]

**Chromosome(s):** 2

**Breakpts/Insertion:** 22A2-3;22D5-E1, 21C8-D1;60D1-2 + 40F;59D4-E1

**Date added:** 9/1/1988 **Donor:** I. Alexandrov

**Comments:** DK2; neutron-induced, dp[DA] penetrant in 50-60% of the population, I.A.; does not delete dp, K.M.; no sign of ds[33k], which in theory is associated with this inversion, K.M. 3/4/01

**Stock Number: 3138**

**Genotype:** Df(2L)b87e25/CyO

**Chromosome(s):** 2

**Breakpts/Insertion:** 34B12-C1;35B10-C1

**Date added:** 5/19/1998 **Donor:** Michael Ashburner

**Comments:** DK2

**Stock Number: 3366**

**Genotype:** y[*]; Df(2L)J2/SM1

**Chromosome(s):** 2

**Breakpts/Insertion:** 31B;32A

**Date added:** 6/1/1989 **Donor:** C. Nusslein-Volhard

**Comments:** DK2; variable Cy expression, K.M. 1/23/97

**Stock Number: 3368**

**Genotype:** Df(2R)cn9/CyO, amos[Roi-1] sp[*] <P>

**Chromosome(s):** 2

**Breakpts/Insertion:** 42E;44C

**Date added:** 6/1/1989 **Donor:** C. Nusslein-Volhard

**Comments:** DK2; poor Cy expression in stock, but seen in some outcrosses, K.C. 5/27/99

**Stock Number: 3467**

**Genotype:** Df(2R)AA21, c[1] px[1] sp[1]/SM1

**Chromosome(s):** 2

**Breakpts/Insertion:** 56F9-17;57D11-12, 56D-E;58E-F (In)

**Date added:** 11/1/1990 **Donor:** Kim Fetchel **Donor's source:** Janis O'Donnell

**Comments:** DK2

**Stock Number: 3518**

**Genotype:** w[a] N[fa-g]; Df(2R)Jp1/CyO

**Chromosome(s):** 1;2

**Breakpts/Insertion:** 51D3-8;52F5-9

**Date added:** 6/12/1989 **Donor:** Bill Saxton

**Comments:** DK2

**Stock Number: 3520**

**Genotype:** w[a] N[fa-g]; Df(2R)Jp8, w[+]/CyO

**Chromosome(s):** 1;2

**Breakpts/Insertion:** 52F5-9;52F10-53A1

**Date added:** 6/12/1989 **Donor:** Bill Saxton

**Comments:** DK2

**Stock Number: 3588**

**Genotype:** Df(2L)TE35BC-24, b[1] pr[1] pk[1] cn[1] sp[1]/CyO

**Chromosome(s):** 2

**Breakpts/Insertion:** 35B4-6;35F1-7

**Date added:** 7/24/1997 **Donor:** Umea Stock Center

**Comments:** DK2; y[1] w[1] floating, U.D.S.C.

**Stock Number: 3591**

**Genotype:** w[1]; Df(2R)Np5, In(2LR)w45-32n, cn[1]/CyO

**Chromosome(s):** 1;2

**Breakpts/Insertion:** 44F10;45D9-E1, 31B;45D9-E1

**Date added:** 7/24/1997 **Donor:** Umea Stock Center

**Comments:** DK2

**Stock Number: 3638**

**Genotype:** Df(2L)net-PMF/SM6a

**Chromosome(s):** 2

**Breakpts/Insertion:** 21A1;21B7-8

**Date added:** 9/1/1990 **Donor:** Jim Kennison

**Comments:** DK2

**Stock Number: 3909**

**Genotype:** w[*]; Df(2R)59AD/SM1

**Chromosome(s):** 1;2

**Breakpts/Insertion:** 59A1-3;59D1-4

**Date added:** 10/3/1997 **Donor:** Christian Lehner

**Comments:** DK2; deficiency apparently uncovers a Minute, C.L.

**Stock Number: 4956**

**Genotype:** Df(2L)XE-3801/CyO, P{ry[+t7.2]=sevRas1.V12}FK1

**Chromosome(s):** 2

**Breakpts/Insertion:** 27E2;28D1

**Date added:** 12/10/1998 **Donor:** Berkeley Drosophila Genome Proj.

**Comments:** DK2

**Stock Number: 4959**

**Genotype:** Df(2L)C'/CyO

**Chromosome(s):** 2

**Breakpts/Insertion:** 40h35;40h38L

**Date added:** 12/10/1998 **Donor:** Berkeley Drosophila Genome Proj.

**Comments:** DK2; Balancer is a guess, K.C. 12/10/98

**Stock Number: 4960**

**Genotype:** Df(2R)CB21/CyO; ry[506]

**Chromosome(s):** 2;3

**Breakpts/Insertion:** 48E;49A

**Date added:** 12/10/1998 **Donor:** Kate Beckingham

**Comments:** DK2

**Stock Number: 4961**

**Genotype:** Df(2R)Kr10, b[1] pr[1] Bl[1] c[1]/CyO

**Chromosome(s):** 2

**Breakpts/Insertion:** 60F1;60F5

**Date added:** 12/10/1998 **Donor:** Berkeley Drosophila Genome Proj.

**Comments:** DK2

**Stock Number: 4966**

**Genotype:** w[1]; Df(2R)w45-30n, cn[1]/CyO

**Chromosome(s):** 1;2

**Breakpts/Insertion:** 45A6-7;45E2-3

**Date added:** 12/10/1998 **Donor:** Berkeley Drosophila Genome Proj.

**Comments:** DK2

**Stock Number: 5246**

**Genotype:** Df(2R)Egfr5, b[1] pr[1] cn[1] sca[1]/CyO, P{ry[+t7.2]=sevRas1.V12}FK1

**Chromosome(s):** 2

**Breakpts/Insertion:** 57D2-8;58D1

**Date added:** 7/13/1999 **Donor:** Jim Price

**Comments:** DK2; rebalanced and returned to collection, w[-] floating, K.M. 2/9/01

**Stock Number: 5330**

**Genotype:** Df(2L)ed1/CyO; P{ry[+t7.2]=ftz/lacC}1

**Chromosome(s):** 2;3

**Breakpts/Insertion:** 24A2;24D4

**Date added:** 8/10/1999 **Donor:** Yash Hiromi

**Comments:** DK2; Deficiency removes slp1 and slp2, Y.H.

**Stock Number: 5420**

**Genotype:** w[*]; Df(2L)Dwee1-W05/CyO; P{ry[+t7.2]=ftz/lacC}1

**Chromosome(s):** 2;3

**Breakpts/Insertion:** 27C2-3;27C4-5

**Date added:** 10/14/1999 **Donor:** Berkeley Drosophila Genome Proj.

**Comments:** DK2; #3571 uncovers four additional genes, but Fs complicates use of the stock, K.C. 5/12/03

**Stock Number: 5574**

**Genotype:** y[1] w[67c23]; Df(2R)k10408, P{w[+mC]=lacW}mthl3[k10408] CG4827[k10408]/CyO

**Chromosome(s):** 1;2

**Breakpts/Insertion:** 54B16;54B16 (Df); 54B16, 2R:13366670..13366670 (R5 flank)

**Date added:** 2/4/2000 **Donor:** Berkeley Drosophila Genome Proj. **Donor's source:** Istvan Kiss

**Comments:** DK2

**Stock Number: 5680**

**Genotype:** Df(2R)robl-c/CyO, y[+]

**Chromosome(s):** 2

**Breakpts/Insertion:** 54B17-C4;54C1-4

**Date added:** 4/19/2000 **Donor:** Aaron Bowman

**Comments:** DK2; Cytology estimated from molecular and complementation data; left breakpoint to the right of l(2)k08901; right breakpoint overlaps Df(2R)k10408, K.C. 3/00 Retains P{w[+mC]=lacW} sequences at end of Df, K.C. 8/01.

**Stock Number: 5869**

**Genotype:** Df(2L)FCK-20, dp[ov1] bw[1]/CyO, P{ry[+t7.2]=sevRas1.V12}FK1

**Chromosome(s):** 2;3

**Breakpts/Insertion:** 32D1;32F1-3

**Date added:** 11/6/2000 **Donor:** Fotis Kafatos

**Comments:** DK2; w[-] floating, K.M. 2/9/01; Cy expression poor, use rough eye as marker, L.J. 8/12/08

**Stock Number: 5879**

**Genotype:** Df(2R)BSC3, w[+mC] unch[k15501] cn[1] bw[1] sp[1]/SM6a, bw[k1]

**Chromosome(s):** 2

**Breakpts/Insertion:** 48E12-F4;49A11-B6 (observed cytology)

**Date added:** 11/6/2000 **Donor:** Kevin Cook

**Comments:** DK2

**Stock Number: 6283**

**Genotype:** Df(2L)BSC4, w[+mC], net[1] cn[1]/SM5

**Chromosome(s):** 2

**Breakpts/Insertion:** 21B7-C1;21C2-3 (observed cytology)

**Date added:** 6/5/2001 **Donor:** Kevin Cook

**Comments:** DK2

**Stock Number: 6299**

**Genotype:** Df(2L)BSC5, w[+mC]/SM6a

**Chromosome(s):** 2

**Breakpts/Insertion:** 26B1-2;26D1-2 (observed cytology)

**Date added:** 6/5/2001 **Donor:** Kevin Cook

**Comments:** DK2

**Stock Number: 6338**

**Genotype:** Df(2L)BSC6, dp[ov1] cn[1]/SM6a

**Chromosome(s):** 2

**Breakpts/Insertion:** 26D3-E1;26F4-7 (observed cytology)

**Date added:** 8/1/2001 **Donor:** Kevin Cook

**Comments:** DK2

**Stock Number: 6374**

**Genotype:** w[1118]; Df(2L)BSC7/CyO

**Chromosome(s):** 1;2

**Breakpts/Insertion:** 26D10-E1;27C1 (observed cytology)

**Date added:** 11/6/2001 **Donor:** Kevin Cook

**Comments:** DK2

**Stock Number: 6455**

**Genotype:** Df(2R)BSC11/SM6a

**Chromosome(s):** 2

**Breakpts/Insertion:** 50E6-F1;51E2-4 (observed cytology)

**Date added:** 1/22/2002 **Donor:** Kevin Cook

**Comments:** DK2

**Stock Number: 6478**

**Genotype:** Df(2L)BSC17/SM6a

**Chromosome(s):** 2

**Breakpts/Insertion:** 30C3-5;30F1 (observed cytology)

**Date added:** 3/4/2002 **Donor:** Kevin Cook

**Comments:** DK2

**Stock Number: 6507**

**Genotype:** y[1] w[*]; Df(2L)drm-P2, P{w[+mC]=lacW}Pdsw[k10101]/SM6b

**Chromosome(s):** 1;2

**Breakpts/Insertion:** 23F3-4;24A1-2 (Df); 23F3, 2L:3466844..3466844 (R5 flank)

**Date added:** 4/22/2002 **Donor:** Ryan Green

**Comments:** DK2

**Stock Number: 6516**

**Genotype:** Df(2R)BSC18/SM6a

**Chromosome(s):** 2

**Breakpts/Insertion:** 50D1;50D2-7 (observed cytology)

**Date added:** 4/22/2002 **Donor:** Kevin Cook

**Comments:** DK2

**Stock Number: 6608**

**Genotype:** Df(2L)BSC16, net[1] cn[1]/SM6a

**Chromosome(s):** 2

**Breakpts/Insertion:** 21C3-4;21C6-8 (observed cytology)

**Date added:** 7/19/2002 **Donor:** Kevin Cook

**Comments:** DK2; Segregating w[1118], K.C.

**Stock Number: 6609**

**Genotype:** Df(2R)BSC19, cn[1] bw[1]/SM6a

**Chromosome(s):** 2

**Breakpts/Insertion:** 56F12-14;57A4 (observed cytology)

**Date added:** 7/19/2002 **Donor:** Kevin Cook

**Comments:** DK2. Df chromosome retains miniwhite from one of the P insertions used in the Df screen, K.C.

**Stock Number: 6647**

**Genotype:** Df(2R)BSC22/SM6a

**Chromosome(s):** 2

**Breakpts/Insertion:** 56D7-E3;56F9-12 (observed cytology)

**Date added:** 9/13/2002 **Donor:** Kevin Cook

**Comments:** DK2

**Stock Number: 6648**

**Genotype:** Df(2L)dpp[d14]/In(2LR)Gla, wg[Gla-1]

**Chromosome(s):** 2

**Breakpts/Insertion:** 22E4-F2;22F3-23A1

**Date added:** 9/13/2002 **Donor:** Kwang Choi

**Comments:** DK2

**Stock Number: 6779**

**Genotype:** y[1] w[67c23]; Df(2R)14H10Y-53/SM6a

**Chromosome(s):** 1;2

**Breakpts/Insertion:** 54D1-2;54E5-7

**Date added:** 12/24/2002 **Donor:** Bill Gelbart

**Comments:** DK2; P{y[+t7.7]=3'wHy} associated with end of Df; Hobo element may be mobilized if crossed to H strain, B.G. Cytological breakpoints estimated from molecular breakpoints, K.C.

**Stock Number: 6780**

**Genotype:** y[1] w[67c23]; Df(2R)14H10W-35/SM6a

**Chromosome(s):** 1;2

**Breakpts/Insertion:** 54E5-7;55B5-7

**Date added:** 12/24/2002 **Donor:** Bill Gelbart

**Comments:** DK2; P{w[+mC]=5'wHy} associated with end of Df; Hobo element may be mobilized if crossed to H strain, B.G. Cytological breakpoints estimated from molecular breakpoints, K.C.

**Stock Number: 6866**

**Genotype:** Df(2R)BSC26/CyO

**Chromosome(s):** 2

**Breakpts/Insertion:** 56C4;56D6-10 (observed cytology)

**Date added:** 12/24/2002 **Donor:** Kevin Cook

**Comments:** DK2; Df chromosome carries miniwhite marker. May be segregating w[*], K.C.

**Stock Number: 6875**

**Genotype:** Df(2L)BSC28/SM6a, bw[k1]

**Chromosome(s):** 2

**Breakpts/Insertion:** 23C5-D1;23E2 (observed cytology)

**Date added:** 2/20/2003 **Donor:** Kevin Cook

**Comments:** DK2; The deficiency chromosome retains the miniwhite marker from P{EP}EP2297 used in screen, K.C. 5/03.

**Stock Number: 6917**

**Genotype:** Df(2R)BSC29, cn[1] bw[1] sp[1]/CyO

**Chromosome(s):** 2

**Breakpts/Insertion:** 45D3-4;45F2-6 (observed cytology)

**Date added:** 4/8/2003 **Donor:** Kevin Cook

**Comments:** DK2. May be segregating w[1118], K.C.

**Stock Number: 6965**

**Genotype:** Df(2L)BSC31, net[1] cn[1]/CyO, b[81f2] rk[81f2]

**Chromosome(s):** 2

**Breakpts/Insertion:** 23E5;23F4-5 (observed cytology)

**Date added:** 4/8/2003 **Donor:** Kevin Cook

**Comments:** DK2. Df(2L)BSC31 retains w[+mC] from P{EP}EP2297 used in the screen, K.C.

**Stock Number: 6999**

**Genotype:** Df(2L)BSC30/SM6a, bw[k1]

**Chromosome(s):** 2

**Breakpts/Insertion:** 34A3;34B7-9 (observed cytology)

**Date added:** 5/2/2003 **Donor:** Kevin Cook

**Comments:** DK2

**Stock Number: 7142**

**Genotype:** Df(2L)BSC32/SM6a, bw[k1]

**Chromosome(s):** 2

**Breakpts/Insertion:** 32A1-2;32C5-D1 (observed cytology)

**Date added:** 7/17/2003 **Donor:** Kevin Cook

**Comments:** DK2; Df(2L)BSC32 retains w[+mC] from P{lacW}l(2)k05812[k05812] used in the screen, K.C.

**Stock Number: 7143**

**Genotype:** Df(2L)BSC36/SM6a, bw[k1]

**Chromosome(s):** 2

**Breakpts/Insertion:** 32D1;32D4-E1 (observed cytology)

**Date added:** 7/17/2003 **Donor:** Kevin Cook

**Comments:** DK2

**Stock Number: 7144**

**Genotype:** Df(2L)BSC37/CyO

**Chromosome(s):** 2

**Breakpts/Insertion:** 22D2-3;22F1-2 (observed cytology)

**Date added:** 9/26/2003 **Donor:** Kevin Cook

**Comments:** DK2. Df chromosome retains mini-white marker from P{EP}EP2232 used in screen. May be segregating w[1118], K.C.

**Stock Number: 7145**

**Genotype:** Df(2R)BSC39, cn[1] bw[1]/SM6a, bw[k1]

**Chromosome(s):** 2

**Breakpts/Insertion:** 48C5-D1;48D5-E1 (observed cytology)

**Date added:** 9/26/2003 **Donor:** Kevin Cook

**Comments:** DK2

**Stock Number: 7146**

**Genotype:** Df(2R)BSC40/SM6a

**Chromosome(s):** 2

**Breakpts/Insertion:** 48E1-2;48E2-10 (observed cytology)

**Date added:** 9/26/2003 **Donor:** Kevin Cook

**Comments:** DK2. The deficiency chromosome retains the miniwhite marker from P{GT1}Rep1[BG01033] used in screen, K.C. 10/04.

**Stock Number: 7147**

**Genotype:** Df(2L)BSC41, dp[ov1] cn[1]/CyO

**Chromosome(s):** 2

**Breakpts/Insertion:** 28A4-B1;28D3-9 (observed cytology)

**Date added:** 9/26/2003 **Donor:** Kevin Cook

**Comments:** DK2. The Df chromosome retains the mini-white marker from one or both P element insertions used in its isolation, K.C. 9/03

**Stock Number: 7273**

**Genotype:** Df(2R)vir130/CyO

**Chromosome(s):** 2

**Breakpts/Insertion:** 59B;59D8-E1

**Date added:** 9/26/2003 **Donor:** Terry Orr-Weaver

**Comments:** DK2; Cytological breakpoints estimated from complementation data, K.C.

**Stock Number: 7414**

**Genotype:** Df(2R)BSC44/SM6a

**Chromosome(s):** 2

**Breakpts/Insertion:** 54B1-2;54B7-10 (observed cytology)

**Date added:** 12/10/2003 **Donor:** Kevin Cook

**Comments:** DK2. The deficiency chromosome retains the miniwhite marker from P{lacW}l(2)k04222b[k04222b] and/or P{EP}CG14478[EP2283] used in screen, K.C. 10/04.

**Stock Number: 7441**

**Genotype:** Df(2R)BSC45, w[+mC]/SM6a

**Chromosome(s):** 2

**Breakpts/Insertion:** 54C8-D1;54E2-7 (observed cytology)

**Date added:** 2/6/2004 **Donor:** Kevin Cook

**Comments:** DK2. Df chromosome retains miniwhite from one of the P element insertions used in the Df screen, K.C.

**Stock Number: 7445**

**Genotype:** Df(2R)BSC49/SM6a

**Chromosome(s):** 2

**Breakpts/Insertion:** 53D9-E1;54B5-10 (observed cytology)

**Date added:** 2/6/2004 **Donor:** Kevin Cook

**Comments:** DK2. The deficiency chromosome retains the miniwhite marker from P{EP}EP2344 and/or P{EP}CG14478[EP2283] used in screen, K.C. 10/04.

**Stock Number: 7497**

**Genotype:** w[1118]; Df(2L)Exel6011, P{w[+mC]=XP-U}Exel6011/CyO

**Chromosome(s):** 1;2

**Breakpts/Insertion:** 25C8;25D5, 2L:5147258;5305646 (R3 author statement->R5)

**Date added:** 3/10/2004 **Donor:** Exelixis, Inc.

**Comments:** DK2. May be segregating TM6B, Tb[1], K.C. 9/05.

**Stock Number: 7531**

**Genotype:** w[1118]; Df(2L)Exel6049, P{w[+mC]=XP-U}Exel6049/CyO

**Chromosome(s):** 1;2

**Breakpts/Insertion:** 40A5;40D3, 2L:21828252;22019296 (R5 flank;R3 author statement->R5)

**Date added:** 3/10/2004 **Donor:** Exelixis, Inc.

**Comments:** DK2

**Stock Number: 7875**

**Genotype:** w[1118]; Df(2R)Exel7130/CyO

**Chromosome(s):** 1;2

**Breakpts/Insertion:** 50D4;50E4, 2R:9960585;10100288 (R5 flank)

**Date added:** 3/30/2004 **Donor:** Exelixis, Inc.

**Comments:** DK2

**Stock Number: 7876**

**Genotype:** w[1118]; Df(2R)Exel7131/CyO

**Chromosome(s):** 1;2

**Breakpts/Insertion:** 50E4;50F6, 2R:10118170--10118172;10247930--10247931 (R5 flank)

**Date added:** 3/30/2004 **Donor:** Exelixis, Inc.

**Comments:** DK2

**Stock Number: 7896**

**Genotype:** w[1118]; Df(2R)Exel7162/CyO

**Chromosome(s):** 1;2

**Breakpts/Insertion:** 56F11;56F16, 2R:16132691--16132995;16201140 (R5 flank)

**Date added:** 3/30/2004 **Donor:** Exelixis, Inc.

**Comments:** DK2

**Stock Number: 8469**

**Genotype:** Df(2L)BSC50/SM6a

**Chromosome(s):** 2

**Breakpts/Insertion:** 30F5;31B1 (R5 estimated cytology), 2L:9984170;10200998 (R5 flank;R5 flank)

**Date added:** 5/31/2004 **Donor:** Kevin Cook

**Comments:** DK2. The deficiency chromosome retains the miniwhite marker from P{EP}CG13130[EP2238] and/or P{EPgy2}EY03684 used in screen, K.C. 10/04.

**Stock Number: 8672**

**Genotype:** w[1118]; Df(2L)BSC106/CyO

**Chromosome(s):** 1;2

**Breakpts/Insertion:** 21B7;21C2, 2L:291728--291846;417947 (R5 flank)

**Date added:** 4/5/2005 **Donor:** Kevin Cook

**Comments:** DK2

**Stock Number: 8674**

**Genotype:** w[1118]; Df(2L)BSC109/CyO

**Chromosome(s):** 1;2

**Breakpts/Insertion:** 25C4;25C8, 2L:5073453;5145500 (R5 flank;R3 author statement->R5)

**Date added:** 4/5/2005 **Donor:** Kevin Cook

**Comments:** DK2

**Stock Number: 8835**

**Genotype:** w[1118]; Df(2L)BSC110/CyO

**Chromosome(s):** 1;2

**Breakpts/Insertion:** 25C1;25C4, 2L:5029595;5064620 (R5 flank)

**Date added:** 6/8/2005 **Donor:** Kevin Cook

**Comments:** DK2

**Stock Number: 8836**

**Genotype:** w[1118]; Df(2L)BSC111/CyO

**Chromosome(s):** 1;2

**Breakpts/Insertion:** 28F5;29B1, 2L:8240266;8362842 (R5 flank;R3 author statement->R5)

**Date added:** 6/8/2005 **Donor:** Kevin Cook

**Comments:** DK2

**Stock Number: 9069**

**Genotype:** w[1118]; Df(2R)ED4065, P{w[+mW.Scer\FRT.hs3]=3'.RS5+3.3'}ED4065/SM6a

**Chromosome(s):** 1;2

**Breakpts/Insertion:** 60C8;60E8, 2R:20290189;20830362 (R5 author statement)

**Date added:** 9/7/2005 **Donor:** DrosDel Project

**Comments:** DK2

**Stock Number: 9270**

**Genotype:** w[1118]; Df(2L)ED250, P{w[+mW.Scer\FRT.hs3]=3'.RS5+3.3'}ED250/SM6a

**Chromosome(s):** 1;2

**Breakpts/Insertion:** 24F4;25A7, 2L:4477085;4821294 (R5 author statement)

**Date added:** 11/20/2005 **Donor:** DrosDel Project

**Comments:** DK2

**Stock Number: 9298**

**Genotype:** w[1118]; Df(2L)ED611, P{w[+mW.Scer\FRT.hs3]=3'.RS5+3.3'}ED611/SM6a

**Chromosome(s):** 1;2

**Breakpts/Insertion:** 29B4;29C3, 2L:8382851;8419818 (R5 author statement)

**Date added:** 11/20/2005 **Donor:** DrosDel Project

**Comments:** DK2

**Stock Number: 9410**

**Genotype:** w[1118]; Df(2R)BSC132/SM6a

**Chromosome(s):** 1;2

**Breakpts/Insertion:** 45F6;46B4, 2R:5482319--5482429;5748332 (R5 flank)

**Date added:** 12/22/2005 **Donor:** Kevin Cook

**Comments:** DK2. Df not in isogenic background of the Exelixis insertion stocks, K.C. 1/07.

**Stock Number: 9496**

**Genotype:** Df(2R)BSC134/Dp(2;3)Cam14T

**Chromosome(s):** 2;3

**Breakpts/Insertion:** 50E1;50E6, 2R:10063602..10063603;10153305..10153306 (R5 flank); 49A;51EF (Dp)

**Date added:** 4/14/2006 **Donor:** Kevin Cook

**Comments:** DK2. Df(2R)BSC134 deletes RpS23, so Df/+ flies show a severe Minute phenotype. This stock does not share the isogenic background of the other BSC and Exel deletions. May be segregating w[1118] and/or cn[1], K.C.

**Stock Number: 9502**

**Genotype:** w[1118]; Df(2L)BSC142/CyO

**Chromosome(s):** 1;2

**Breakpts/Insertion:** 28C3;28D3, 2L:7774037;8012787 (R5 flank)

**Date added:** 4/14/2006 **Donor:** Kevin Cook

**Comments:** DK2

**Stock Number: 9503**

**Genotype:** w[1118]; Df(2L)BSC143/CyO

**Chromosome(s):** 1;2

**Breakpts/Insertion:** 31B1;31D9, 2L:10209408;10333704 (R5 flank)

**Date added:** 4/14/2006 **Donor:** Kevin Cook

**Comments:** DK2

**Stock Number: 9505**

**Genotype:** w[1118]; Df(2L)BSC145/CyO

**Chromosome(s):** 1;2

**Breakpts/Insertion:** 32C1;32C1, 2L:10967405;11001945--11001966 (R5 flank)

**Date added:** 4/14/2006 **Donor:** Kevin Cook

**Comments:** DK2

**Stock Number: 9506**

**Genotype:** w[1118]; Df(2L)BSC147/CyO

**Chromosome(s):** 1;2

**Breakpts/Insertion:** 34C1;34C6, 2L:13445419;13665416--13665417 (R5 flank)

**Date added:** 4/14/2006 **Donor:** Kevin Cook

**Comments:** DK2

**Stock Number: 9510**

**Genotype:** w[1118]; Df(2L)BSC151/CyO

**Chromosome(s):** 1;2

**Breakpts/Insertion:** 40A5;40E5, 2L:21828581;22139023 (R5 flank;R3 author statement->R5)

**Date added:** 4/14/2006 **Donor:** Kevin Cook

**Comments:** DK2

**Stock Number: 9594**

**Genotype:** Df(2L)BSC159/Dp(2;2)Cam5, bw[1] sp[1]

**Chromosome(s):** 2

**Breakpts/Insertion:** 34B4;34C4, 2L:13290761..13290762;13536086 (R5 flank); 32F;35B1-2 (Dp)

**Date added:** 9/1/2006 **Donor:** Kevin Cook

**Comments:** DK2. Df associated with a Minute phenotype from deletion of RpL24; stock does not retain the Exelixis isogenic background; may be segregating w[1118], K.C.

**Stock Number: 9596**

**Genotype:** w[1118]; Df(2R)BSC161/CyO

**Chromosome(s):** 1;2

**Breakpts/Insertion:** 54B2;54B17, 2R:13192288;13372333 (R5 flank)

**Date added:** 9/1/2006 **Donor:** Kevin Cook

**Comments:** DK2

**Stock Number: 9691**

**Genotype:** w[1118]; Df(2R)BSC155/CyO-Df(2R)B80, y[+]

**Chromosome(s):** 1;2

**Breakpts/Insertion:** 60B8;60C4, 2R:19968928;20145426 (R5 flank)

**Date added:** 12/14/2006 **Donor:** Kevin Cook

**Comments:** DK2. This stock does not retain the isogenic background common to other BSC and Exel deletions, K.C.

**Stock Number: 25078**

**Genotype:** w[1118]; Df(2R)BSC550/CyO

**Chromosome(s):** 1;2

**Breakpts/Insertion:** 53C1;53C6, 2R:12224286;12382237 (R5 flank)

**Date added:** 7/18/2008 **Donor:** Kevin Cook

**Comments:** DK2
