# Supplementary material for: Use of Mutagenesis, Genetic Mapping and Next Generation Transcriptomics to Investigate Insecticide Resistance Mechanisms
Source: PLoS One. 2012 Jun 29;7(6):e40296. doi: 10.1371/journal.pone.0040296 (PMC3386967; doi:10.1371/journal.pone.0040296)
Supplement: Text S2 — P element stocks. (DOC) [file pone.0040296.s002.doc]

P element stocks:

**Stock Number: 13763**

**Genotype:** y[1] w[67c23]; P{y[+mDint2] w[BR.E.BR]=SUPor-P}Arf51F[KG02753]

**Chromosome(s):** 1;2

**Breakpts/Insertion:** 51F6, 2R:11210503..11210503 (R5)

**Date added:** 4/5/2002 **Donor:** Berkeley Drosophila Genome Proj. **Donor's source:** Hugo J. Bellen

**Comments:** May be segregating CyO and ry[506], K.C. 5/03

**Stock Number: 13840**

**Genotype:** y[1] w[67c23]; P{y[+mDint2] w[BR.E.BR]=SUPor-P}KG04715

**Chromosome(s):** 1;2

**Breakpts/Insertion:** 47B4, 2R:6560770..6560770 (R5)

**Date added:** 4/5/2002 **Donor:** Berkeley Drosophila Genome Proj. **Donor's source:** Hugo J. Bellen

**Comments:** May be segregating CyO and ry[506], K.C. 5/03

**Stock Number: 14341**

**Genotype:** y[1] w[67c23]; P{y[+mDint2] w[BR.E.BR]=SUPor-P}CAP[KG00083]

**Chromosome(s):** 1;2

**Breakpts/Insertion:** 47A1, 2R:6189895..6189895 (R5 flank)

**Date added:** 6/20/2002 **Donor:** Berkeley Drosophila Genome Proj. **Donor's source:** Gary Karpen

**Comments:** C(1;Y)1, y[1] may be segregating, B.D.G.P. May be segregating CyO and ry[506], K.C. 7/03

**Stock Number: 12973**

**Genotype:** y[1] w[67c23]; P{y[+mDint2] w[BR.E.BR]=SUPor-P}KG01086

**Chromosome(s):** 1;2

**Breakpts/Insertion:** 41B1, 2R:504496..504496 (R5 flank)

**Date added:** 3/14/2002 **Donor:** Berkeley Drosophila Genome Proj. **Donor's source:** Hugo J. Bellen

**Comments:** May be segregating CyO and ry[506], K.C. 5/03
